# Light-mediated control of gene expression in the anoxygenic phototrophic bacterium *Rhodobacter capsulatus* using photocaged inducers

**DOI:** 10.3389/fbioe.2022.902059

**Published:** 2022-09-30

**Authors:** Fabienne Hilgers, Fabian Hogenkamp, Oliver Klaus, Luzie Kruse, Anita Loeschcke, Claus Bier, Dennis Binder, Karl-Erich Jaeger, Jörg Pietruszka, Thomas Drepper

**Affiliations:** ^1^ Institute of Molecular Enzyme Technology, Heinrich Heine University Düsseldorf at Forschungszentrum Jülich, Jülich, Germany; ^2^ Institute of Bioorganic Chemistry, Heinrich Heine University Düsseldorf at Forschungszentrum Jülich, Jülich, Germany; ^3^ Institute of Bio- and Geosciences: Biotechnology (IBG-1), Forschungszentrum Jülich, Jülich, Germany

**Keywords:** caged compounds, light-controlled gene expression, optogenetics, purple non-sulfur photosynthetic bacteria, *Rhodobacter capsulatus*

## Abstract

Photocaged inducer molecules, especially photocaged isopropyl-β-d-1-thiogalactopyranoside (cIPTG), are well-established optochemical tools for light-regulated gene expression and have been intensively applied in *Escherichia coli* and other bacteria including *Corynebacterium glutamicum, Pseudomonas putida* or *Bacillus subtilis*. In this study, we aimed to implement a light-mediated on-switch for target gene expression in the facultative anoxygenic phototroph *Rhodobacter capsulatus* by using different cIPTG variants under both phototrophic and non-phototrophic cultivation conditions. We could demonstrate that especially 6-nitropiperonyl-(NP)-cIPTG can be applied for light-mediated induction of target gene expression in this facultative phototrophic bacterium. Furthermore, we successfully applied the optochemical approach to induce the intrinsic carotenoid biosynthesis to showcase engineering of a cellular function. Photocaged IPTG thus represents a light-responsive tool, which offers various promising properties suitable for future applications in biology and biotechnology including automated multi-factorial control of cellular functions as well as optimization of production processes.

## Graphical Abstract

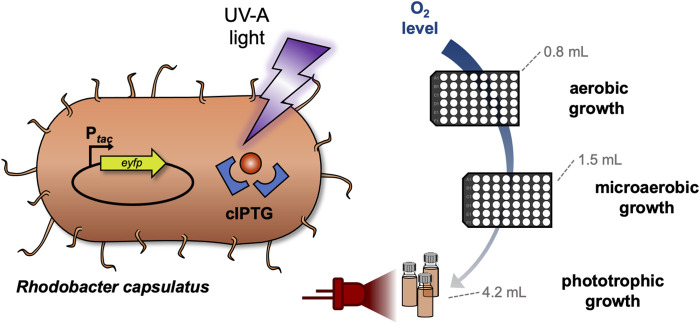

## Introduction

In the field of optogenetics, the application of light offers various advantageous properties such as non-invasive control with high spatiotemporal resolution (Deiters, 2009; Drepper et al., 2011; Brieke et al., 2012; Gardner and Deiters, 2012; Bardhan and Deiters, 2019; Baumschlager and Khammash, 2021). In this context, photo-labile protecting groups are useful for controlling a multitude of cellular processes including cell signaling (Liu et al., 2017; Bardhan and Deiters, 2019; Kolarski et al., 2019) or gene expression (Young and Deiters, 2007; Gardner et al., 2011; Binder et al., 2014; Bier et al., 2016; Kusen et al., 2016; Hogenkamp et al., 2022). For light-regulated gene expression, especially photocaged isopropyl-β-d-1-thiogalactopyranoside (cIPTG) was applied, e.g., for automated optimization of heterologous gene expression in *Escherichia coli* using a high-throughput screening system (Wandrey et al., 2016). The growth medium can be supplemented with photocaged IPTG, which remains non-functional, until a light-pulse releases the inducer from its caging group so that it can induce expression of target genes under control of a *lac* (or *lac*-type) promoter by specific interaction with the LacI repressor. Photocaged IPTG has further been utilized to control gene expression in the Gram-positive bacteria *Corynebacterium glutamicum* (Binder et al., 2016; Burmeister et al., 2021) and *Bacillus subtilis* as well as the Gram-negative bacterium *Pseudomonas putida* (Hogenkamp et al., 2021). However, the applicability of photocaged inducer molecules for light-mediated control of bio(techno)logical processes has not been studied so far in phototrophic bacteria, since the individual emission spectra of applied light sources might hamper the separation of photosynthetic from optochemical control processes. In this study, we thus evaluated if photocaged IPTG can be used to implement non-invasive light control for target gene expression in the facultative anaerobic phototroph *R. capsulatus*.

*R. capsulatus* is a metabolically versatile bacterium that is able to grow either under phototrophic conditions (i.e., in the absence of oxygen and presence of light) by performing anoxygenic photosynthesis or under chemotrophic conditions (in the presence of an electron acceptor, e.g., molecular oxygen) (Stoppani et al., 1955; Tabita, 1995; Strnad et al., 2010). Upon reduction of oxygen tension, *R. capsulatus* starts to form an intracytoplasmic membrane (ICM) system, which harbors the photosynthesis apparatus (Drews and Oelze, 1981; Wu and Bauer, 2008; Tucker et al., 2010; Drews, 2013). Since this ICM system can function as a naturally enlarged storage compartment for membrane-embedded enzymes and metabolites, the phototrophic lifestyle renders novel approaches possible where *R. capsulatus* is applied as an alternative host for the production of otherwise difficult-to-express membrane proteins as well as hydrophobic secondary metabolites such as plant-derived terpenoids (Khan et al., 2015; Loeschcke et al., 2017; Hage-Hülsmann et al., 2019; Troost et al., 2019; Hilgers et al., 2021; Klaus et al., 2022).

**FIGURE 1:**
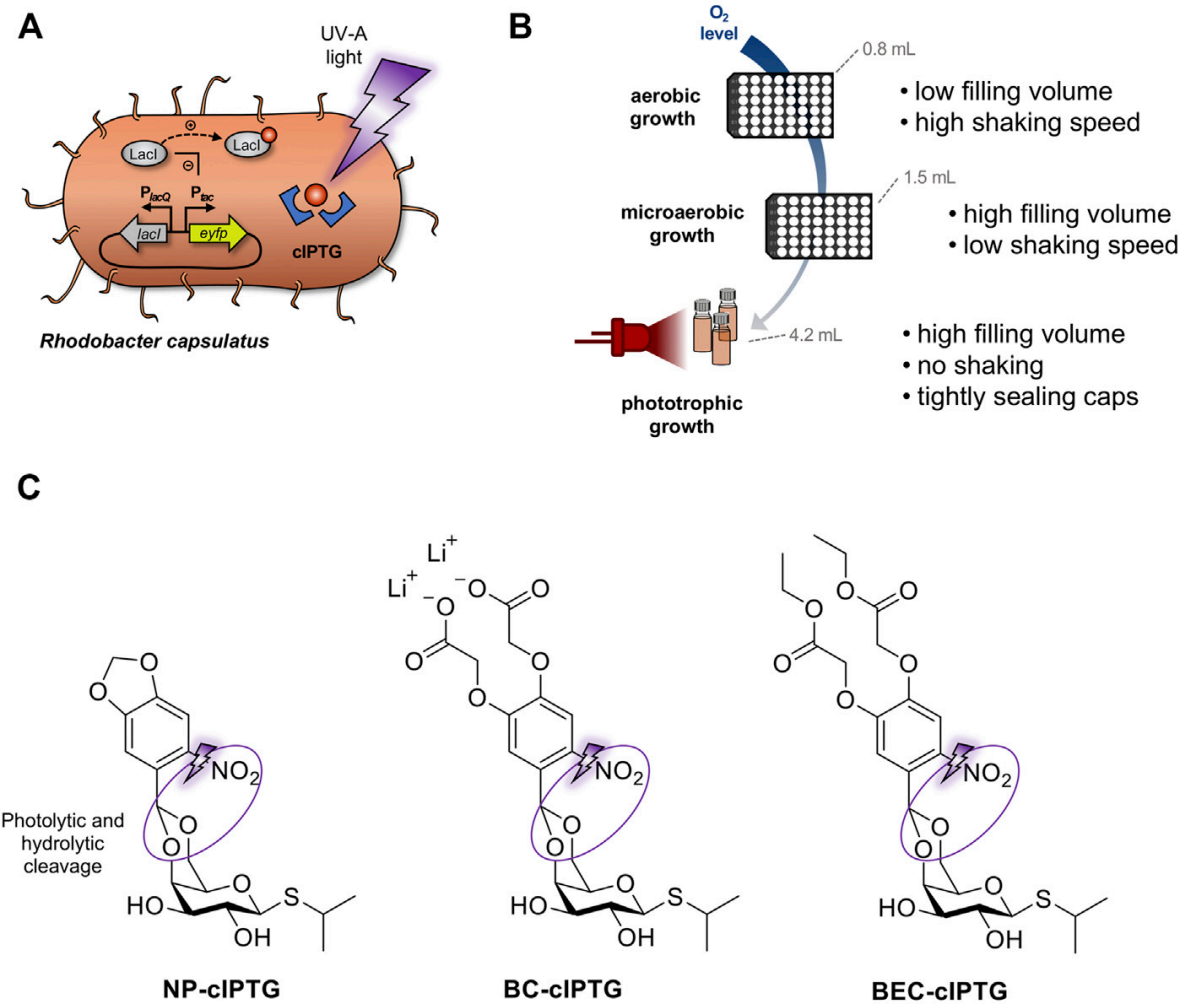
Establishment of optochemical control over gene expression in *R. capsulatus* under non-phototrophic and phototrophic growth conditions. **(A)** Light-controlled expression of the reporter gene *eyfp* in *R. capsulatus* using photocaged IPTG (cIPTG; red circle with blue frames). Upon illumination with UV-A light (purple flash symbol), the protection group is cleaved off, the previously inactive IPTG molecule is released and induces LacI/P_*tac*_-mediated *eyfp* expression. **(B)** For analyzing light-dependent control of gene expression, *R. capsulatus* cells were either cultivated under non-phototrophic conditions (i.e., aerobic or microaerobic conditions in Round Well Plates with filling volumes of 0.8 and 1.5 mL, respectively) or anaerobic, phototrophic growth conditions using NIR light LED as sole light source (red LED diodes; *λ*_max_ = 850 nm, small scale screw neck vials). **(C)** Photocaged IPTG variants NP-cIPTG, BC-cIPTG and BEC-cIPTG used in this study as well as the respective cleavage sites addressed by photolysis and subsequent enzymatic hydrolysis (purple circles).

Phototrophic growth offers robust and relatively fast cell division of *R. capsulatus* cells, as the broad emission spectrum of bulb light or natural daylight is suitable for the excitation of all photopigments, namely the carotenoids spheroidene (λ*^abs^* *_max_* = 454, 478, 509 nm) and spheroidenone (λ*^abs^* *_max_* = 500 nm) as well as bacteriochlorophyll *a* (BChl *a*, λ*^abs^* *_max_* = 800 and 860 nm) (Obeid et al., 2009; Boran et al., 2010; Katzke et al., 2010; Elkahlout et al., 2019). In the presented study, we evaluated whether optochemical induction of gene expression can be established under non-phototrophic and phototrophic growth conditions by using UV-A light-responsive cIPTG. For phototrophic cultivation of *R. capsulatus* cells, cell suspensions were illuminated using near infrared (NIR) light-emitting LEDs (*λ*_max_ = 850 nm) instead of commonly used broad-spectrum bulb light to avoid unintended activation of the optochemical on-switch (Figure 1A).

Since the formation of the ICM system might function as natural, hydrophobic diffusion barrier, which could affect the uptake of caged inducer molecules under phototrophic growth conditions, we additionally evaluated the applicability of the three differentially soluble cIPTG variants 6-nitropiperonyl photocaged IPTG (NP-cIPTG), 4,5-bis(carboxymethoxy)-2-nitrobenzyl photocaged IPTG (BC-cIPTG) and 4,5-bis(ethoxycarbonylmethoxy)-2-nitrobenzyl photocaged IPTG (BEC-cIPTG) (Hogenkamp et al., 2021) for this optochemical approach (Figure 1). While the well-established NP-cIPTG shows a low solubility in the cultivation medium resulting in the formation of an emulsion, BC-cIPTG offers an approximately 200-fold higher water-solubility. In contrast, BEC-cIPTG exhibits low water-solubility but offers hydrophobic side chains that might enhance its ICM-permeability. The original water solubility of IPTG is restored *via* the uncaging process, leading to a dissolution of the emulsion-like mixture after UV-A illumination.

As those photocaged molecules strongly differ in hydrophobicity and water-solubility, we wanted to compare their general usability for light-controlled gene expression in *R. capsulatus* under phototrophic (formation of high ICM levels) and non-phototrophic (moderate to low ICM formation) conditions. In summary, we could demonstrate that photocaged inducer molecules and especially NP-cIPTG can be applied for light-mediated control over gene expression in the phototrophic bacterium *R. capsulatus*. Furthermore, we successfully applied this optochemical approach to control the intrinsic carotenoid pathway to showcase engineering of secondary metabolite biosynthesis. This optochemical on-switch thus offers various promising properties suitable for biotechnological applications in phototrophic hosts including automated bioprocess engineering approaches under defined light conditions.

## Results

### cIPTG-mediated light control of gene expression in *R. capsulatus* under non-phototrophic conditions

**FIGURE 2:**
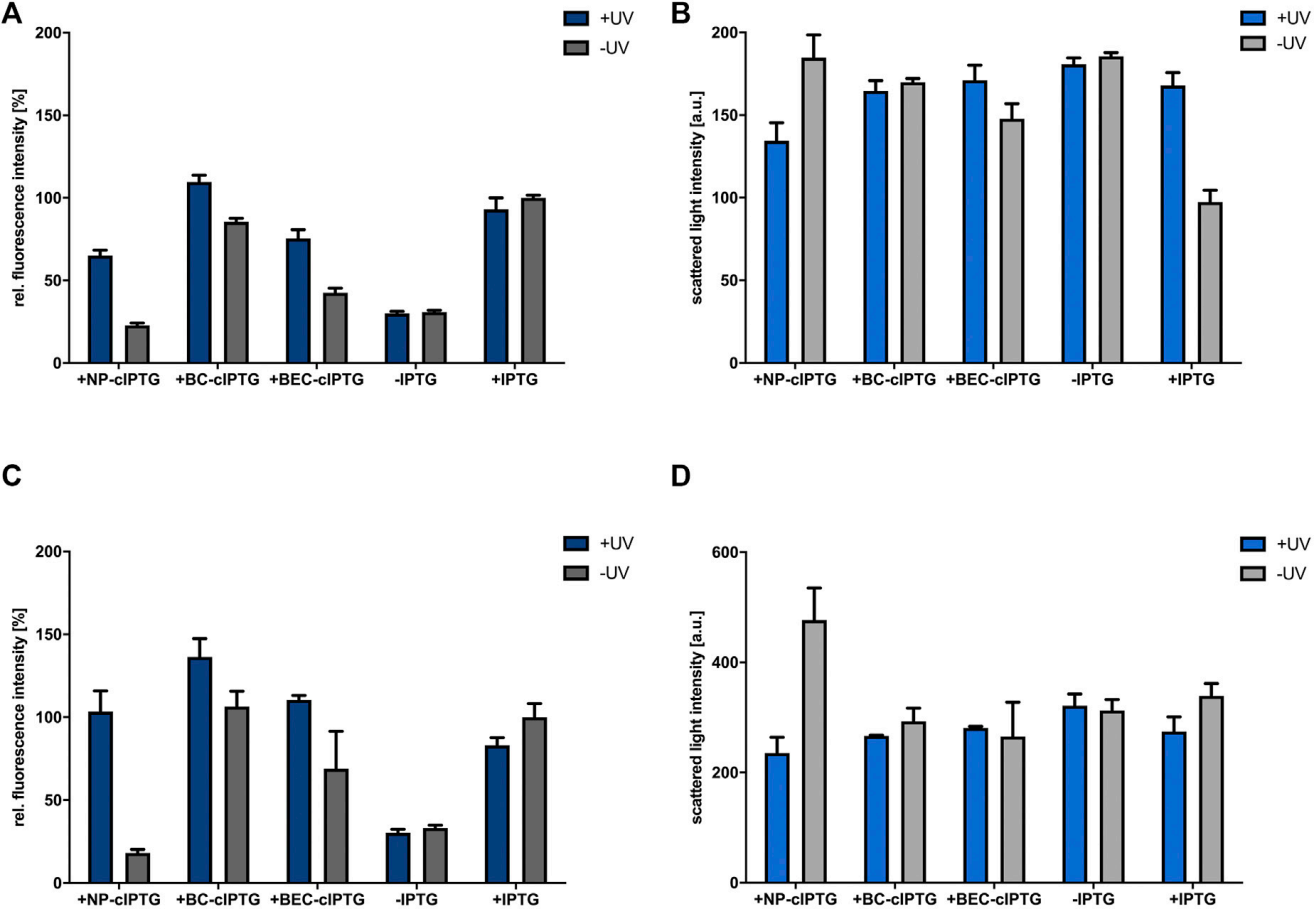
Application of cIPTG derivatives for light-mediated control of gene expression in *R. capsulatus* under non-phototrophic conditions. Light-controlled *eyfp* reporter gene expression and corresponding cell densities in aerobically **(A,B)** and microaerobically **(C,D)** grown *R. capsulatus* SB1003 cultures carrying pRholHi-2-eYFP using the three cIPTG derivatives NP-, BC- and BEC-cIPTG. Biomass-normalized eYFP *in vivo* fluorescence (*λ*_ex_ = 508 nm, *λ*_em_ = 532 nm) and cell growth represented by the scattered light intensity (λ = 620 nm) of cultures supplemented with 1 mm of each cIPTG variant is shown in relation to a control culture (1 mm IPTG) after 48 h of cultivation (RCV medium, 30°C; for aerobic cultures 800 rpm and 800 µL filling volume and for microaerobic cultures 400 rpm and 1,500 µL filling volume). Induction of reporter gene expression was performed after 9 h *via* UV-A light exposure at 365 nm (∼1 mW/cm^2^) for 30 min or the addition of 1 mm IPTG. **(B,D)**. Values are means of individual biological triplicates. Error bars indicate the respective standard deviations.

In order to establish a cIPTG-based optochemical control of gene expression in the facultative phototrophic organism *R. capsulatus*, we first constructed the expression plasmid pRholHi-2-eYFP containing the repressor gene *lacI* and the LacI-controlled P_*tac*_ promoter originating from the shuttle vector pEKEx2 (Eikmanns et al., 1991) (Supplementary Table S1) as well as the downstream located *eyfp* reporter gene, whose expression was first analyzed in *R. capsulatus* under aerobic and microaerobic conditions (i.e., low and intermediate induction of ICM formation) in the dark. Since we performed small scale cultivation of *R. capsulatus* in the BioLector microbioreactor for the first time, the filling volume of the Round Well Plates as well as the shaking frequencies were experimentally determined to adjust the aeration of cultures during non-phototrophic growth (Figure 1B; Supplementary Figure S1). To this end, the *R. capsulatus* strain carrying the corresponding *eyfp* expression vector pRholHi-2-eYFP was cultivated in the dark without the addition of IPTG but with varying filling volumes and shaking frequencies (800 µL and 800 rpm, 1,000 µL or 1,500 μL and 400 rpm) for 48 h at 30°C. In these cultures, bacterial growth (scattered light intensity, Supplementary Figure S1A) and the dissolved oxygen tension (DOT; Supplementary Figure S1B) were online-monitored. Sufficient conditions for aerobic and microaerobic growth were found to be 800 µL filling volume and 800 rpm shaking frequency or 1,500 µL and 400 rpm, respectively. To evaluate the functionality and inducibility of the LacI/P_*tac*_ promoter system in *R. capsulatus* under non-phototrophic growth conditions, IPTG was added at increasing concentrations (0–10 mm) to the medium after 9 h of cultivation (early logarithmic growth phase) and reporter gene expression was analyzed by detecting the specific eYFP fluorescence (Supplementary Figures S2A,B). The results showed a comparatively high *eyfp* expression for IPTG concentrations of 1 mm and above; thus, 1 mm was chosen as sufficient inducer concentration in all further experiments. To evaluate, whether cIPTG derivatives can be applied for light-controlled gene expression in *R. capsulatus* under non-phototrophic conditions, we analyzed the induction response of NP-cIPTG, BC-cIPTG and BEC-cIPTG in comparison to IPTG for the strain *R. capsulatus* SB1003/pRholHi-2-eYFP in the absence and presence of UV-A light (*λ*_max_ = 365 nm, 1 mW/cm^2^, 30 min). Both experiments revealed considerable induction levels for all three cIPTG derivatives leading to induction levels of at least 70% and up to nearly 150% in comparison to IPTG (Figures 2A,C). The corresponding measurements of the respective cell densities (Figures 2B,D) further revealed that cell growth was slightly affected by the respective photolysis products or UV-A light exposure under aerobic conditions and considerably less influenced under microaerobic conditions. This can also be confirmed by the growth curves and fluorescence measurements (Supplementary Figures S4A,B, S5).

However, only NP-cIPTG showed a reasonably low induction level under light exclusion for aerobic and microaerobic cultivation conditions over 48 h in RCV medium at 30°C, whereas the use of BC-cIPTG and BEC-cIPTG led to high illumination-independent fluorescence signals. In order to analyze, if the *Rhodobacter* cultivation medium is responsible for the observed instability effect, the light-independent cleavage of IPTG was analyzed in a control experiment using the well-established strain *E. coli* Tuner (DE3) (Binder et al., 2014). To this end, we measured the induction of *eyfp* reporter gene expression upon addition of cIPTG variants, which were previously incubated either in LB or RCV medium (Supplementary Figure S4C). These data show that no auto-hydrolysis was observed for any cIPTG derivative in sole RCV or LB medium and thus could give a first hint that host specific enzymes or metabolites might be involved in the light-independent hydrolysis of BC- and BEC-cIPTG at least under aerobic and microaerobic growth conditions. We therefore identified the non-toxic and functional NP-cIPTG as the most promising candidate for light-controlled induction of gene expression in *R. capsulatus* under non-phototrophic conditions.

In a next step, we evaluated the applicability of cIPTG as an optochemical on-switch under phototrophic conditions, where additional bottlenecks may arise such as permanent illumination necessary for photosynthesis and the extensive formation of ICM that might function as additional cellular diffusion barriers.

### Light-mediated induction of gene expression in *R. capsulatus* under anaerobic, phototrophic growth conditions

In the laboratory, phototrophic *R. capsulatus* cultures are typically grown in sealed hungate tubes under constant illumination with bulb light to ensure both a strict oxygen exclusion and optimal light conditions. First, we analyzed whether illumination of cultures, which is essential for phototrophic growth, already leads to unwanted uncaging effects. For this purpose, we used 1,2-dimethoxy-4-nitrobenzene (DMNB) as a molecular UV-A light detector suitable for the analysis of nitrobenzyl-based photouncaging processes (Pelliccioli and Wirz, 2002; Nakayama et al., 2011; Brieke et al., 2012). Under UV-A light exposure, the DMNB molecule undergoes a photoconversion into 2-methoxy-5-nitrophenolate (MNP) accompanied by a precisely detectable increase of absorption at *λ*_max_ = 422 nm (Supplementary Figures S7A–C) (Van Riel et al., 1981; Zhang et al., 1999). To detect undesired uncaging processes induced by different light sources applicable for phototrophic cultivation of *R. capsulatus* cells (Kaschner et al., 2014; Peters et al., 2019; Hilgers et al., 2021), the DMNB solution was exposed for 48 h either to bulb light (broad emission spectrum with high NIR light and low UV light components) or NIR light (monochromatic, *λ*_max_ = 850 nm). Additionally, a dark control and samples with increasing UV-A light exposure times from 30 to 90 min were performed (Supplementary Figure S7D).

Interestingly, analysis of photochemical MNP formation at 422 nm after 24 and 48 h revealed that exposure to bulb light leads to absorption values comparable to samples that were irradiated by UV-A light for 30 min. Thus, bulb light is not applicable as a light source for the phototrophic cultivation of *R. capsulatus* if cIPTG is intended to be used as an optochemical on-switch. In contrast, NIR light at 850 nm (i.e., the absorption maximum of the photopigment BChl a) seems to be a promising alternative light source, as even after 48 h no photoconversion of DMNB could be detected in comparison to the dark control. To determine, which NIR light intensities are needed for efficient phototrophic growth of *R. capsulatus,* cells were cultivated with NIR light of increasing intensities ranging from 0.5 mW/cm^2^ up to 5.1 mW/cm^2^ using a custom-made NIR light LED panel (Hilgers et al., 2021) and analyzed the growth behavior (Supplementary Figure S8). The data revealed that both too high and too low NIR light intensities have a negative effect on phototrophic growth due to insufficient energy supply or adverse cultivation temperatures. However, illumination properties of NIR LED panels could be appropriately adjusted for phototrophic growth of *R. capsulatus*: An NIR-light intensity of 1.7 mW/cm^2^ led to similar cell densities as compared to bulb light irradiation without exceeding the optimal growth temperature. It is worth mentioning that an altered color could be observed for *R. capsulatus* cultures grown under NIR light illumination, which might indicate an adaptation of photopigment synthesis or accumulation.

**FIGURE 3:**
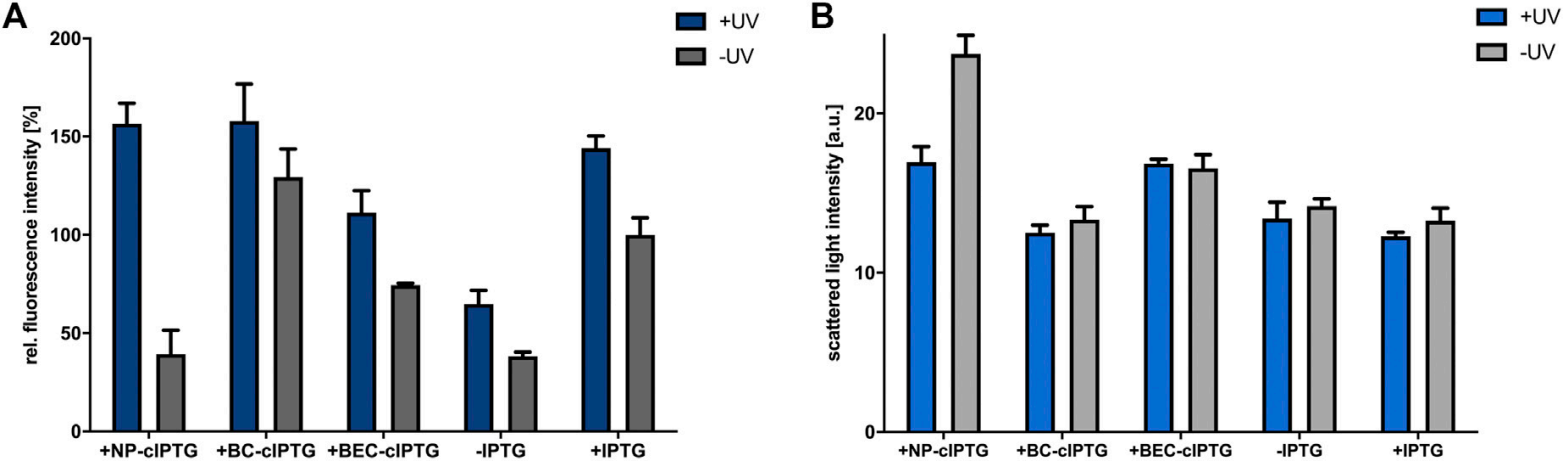
Application of cIPTG derivatives for light-controlled induction of gene expression in phototrophically grown *R. capsulatus* cells. Light-controlled *eyfp* reporter gene expression **(A)** and corresponding cell densities **(B)** of phototrophically grown *R. capsulatus* SB1003 cells carrying pRholHi-2-eYFP using the cIPTG derivatives NP-, BC- and BEC-cIPTG. Biomass-normalized eYFP *in vivo* fluorescence (*λ*_ex_ = 508 nm, *λ*_em_ = 532 nm) and cell growth represented by the scattered light intensity of cultures supplemented with 1 mm of each cIPTG variant are shown in relation to a control culture (1 mm IPTG) after 48 h of cultivation [30°C, screw neck vials, constant NIR light illumination (*λ*_max_ = 850 nm, 1.7 mW/cm^2^)]. Induction of reporter gene expression was performed after 6 h *via* UV-A light exposure (365 nm; ∼2 mW/cm^2^) for 30 min. Values are means of individual biological triplicates. Error bars indicate the respective standard deviations.

Next, we analyzed whether photocleavable cIPTG derivatives can be applied for UV-A light-dependent control of gene expression in phototrophically growing *R. capsulatus* cells. To this end, the strain *R. capsulatus* SB1003 carrying the plasmid pRholHi-2-eYFP was cultivated under constant NIR light illumination and reporter gene expression in cIPTG-supplemented cultures was induced after 6 h (early logarithmic growth phase) with 30 min of UV-A light (365 nm; ∼2 mW/cm^2^). Resulting eYFP fluorescence signals were analyzed when cells reached the stationary growth phase (48 h). As shown in Figure 3A, UV-A light-induced uncaging of the cIPTG derivatives NP- and BC-cIPTG resulted in even higher *eyfp* expression levels as in the control experiment, where conventional IPTG was added to UV-A exposed cultures, while the expression strength of BEC-cIPTG was slightly lower. Remarkably, changing the water solubility and thus the hydrophobicity of cIPTG did not result in improved *eyfp* expression levels under phototrophic growth conditions. Based on the approximately similar fluorescence signals observed after IPTG and NP-cIPTG induction under aerobic, microaerobic, and phototrophic conditions, it can thus be concluded that the putative IPTG diffusion barrier formed by ICM do not exert a significant influence on NP-cIPTG uptake. In addition, as previously observed in cultures that have been grown under aerobic and microaerobic conditions, BC-cIPTG and BEC-cIPTG were not stable during phototrophic cultivation, resulting in almost equally high induction levels in the control (−UV) as compared to the UV-A light exposed cultures (+UV). In contrast, NP-cIPTG showed sufficient stability and neglectable toxicity under phototrophic growth conditions (Figure 3; Supplementary Figures S4,S6).

Further it should be noted that the cell density-normalized eYFP fluorescence under phototrophic conditions is significantly higher than under aerobic or microaerobic conditions (Supplementary Figure S2). Similar results were obtained in the study of Katzke and co-workers, where the protein yield of the T7 expression strain *R. capsulatus* B10S-T7 was superior under phototrophic growth conditions (Katzke et al., 2010). This observation further supports the assumption that an increased energy availability during phototrophic growth might facilitate efficient synthesis of heterologous proteins in *R. capsulatus*. In this context, it is worth mentioning that phototrophically cultivated *R. capsulatus* cells showed an increased eYFP fluorescence in the presence and absence of conventional IPTG, when cultures were additionally illuminated with UV-A light. This observation might indicate that UV-A light illumination, although it was only briefly applied in an early phase of cell cultivation, can also lead to an excitation of the photopigment BChl *a* in the Soret band (Happ et al., 2005; Xin et al., 2007). This might further boost photosynthesis and thereby facilitate energy-demanding processes including *eyfp* over-expression if applied NIR light exposure is not sufficient for full BChl *a* excitation.

Additionally, phototrophically grown cultures without supplemented IPTG exhibited a high basal *eyfp* expression level (Figures 2, 3; Supplementary Figures S2A–C). Since wild-type control cultures without an expression plasmid did not show significantly increased fluorescence levels, the promoter system appears to be affected by high basal expression, possibly caused by an insufficient amount of the LacI repressor. As noted in previous studies, increasing the level of LacI has been shown to be extremely valuable for reducing basal expression and thereby increasing the tightness of an expression system (Dubendorf and Studier, 1991; Studier, 1991).

The data presented so far can be summarized as follows: 1) the *eyfp* expression experiments clearly demonstrated that NP-cIPTG can be used as an optochemical on-switch for light-dependent induction of heterologous gene expression in *R. capsulatus*. 2) Neither constant illumination with NIR light, which is required for photosynthesis, nor the formation of ICM negatively affected the function of light-responsive cIPTG. Hence, this optochemical switch can be used for the facultative phototrophic bacterium under both non-phototrophic and phototrophic growth conditions.

### Optimization of pRholHi-2-eYFP

**FIGURE 4:**
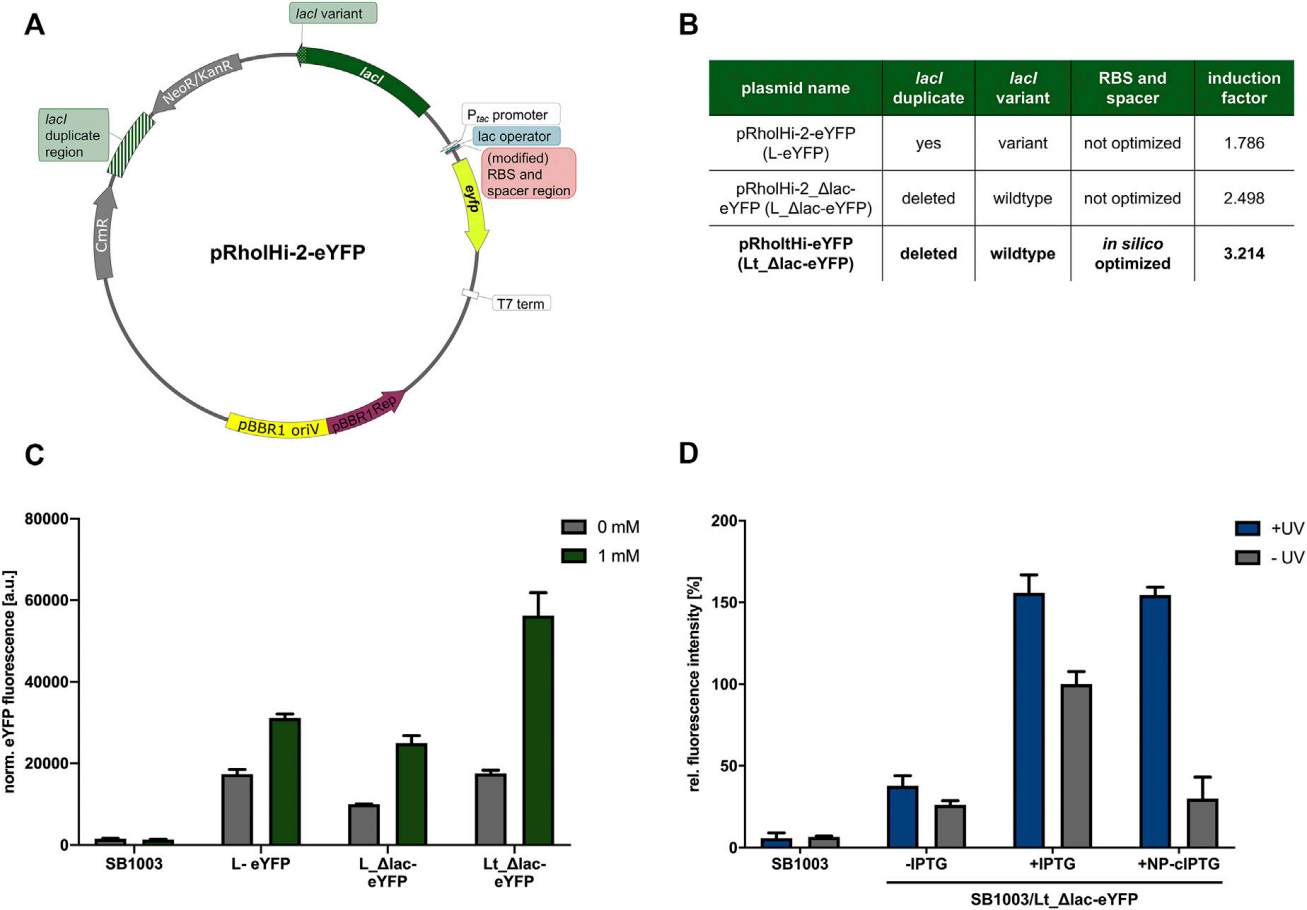
Optimization of expression plasmid pRholHi-2-eYFP. To improve the properties of the expression plasmid pRholHi-2-eYFP **(A)** several engineering steps were conducted **(A,B)**. First, a defective *lacI* gene (*lacI* variant) was exchanged by the corresponding wildtype *lacI* gene from *E. coli* K12. Secondly, a 519-bp *lacI*-homologous DNA fragment approximately 1.5 kb upstream of the *lacI* gene was deleted (*lacI* duplicate region). Additionally, the ribosome binding site (RBS) consisting of the Shine-Dalgarno (SD) sequence, and its corresponding downstream spacer was replaced by an *in silico* optimized version. The induction factors of the respective expression systems were calculated as the ratio of the normalized eYFP fluorescence values of induced and non-induced cultures. **(C)** After construction, the three plasmid variants were evaluated regarding their resulting basal expression levels and induction factors in phototrophically grown *R. capsulatus* SB1003 cultures. Biomass-normalized eYFP *in vivo* fluorescence (*λ*_ex_ = 508 nm, *λ*_em_ = 532 nm) of cultures supplemented with 1 mm IPTG is shown in comparison to uninduced cultures and the *R. capsulatus* wildtype control strain after 48 h of cultivation (30°C, screw neck vials, NIR light: *λ*_max_ = 850 nm, 1.7 mW/cm^2^). Induction of reporter gene expression was performed after 6 h. **(D)** Light-controlled *eyfp* reporter gene expression in phototrophically grown *R. capsulatus* SB1003 cells carrying the best performing expression plasmid pRholtHi-eYFP using NP-cIPTG. Biomass-normalized eYFP *in vivo* fluorescence (*λ*_ex_ = 508 nm, *λ*_em_ = 532 nm) of cultures supplemented with 1 mm NP-cIPTG is shown in relation to a 1 mm IPTG and a *R. capsulatus* wildtype control after 48 h of cultivation (30°C, screw neck vials, NIR light: *λ*_max_ = 850 nm, 1.7 mW/cm^2^). Induction of reporter gene expression was performed after 6 h *via* UV-A light exposure (365 nm; ∼2 mW/cm^2^, 30 min). Values are means of individual biological triplicates. Error bars indicate the respective standard deviations.

**FIGURE 5:**
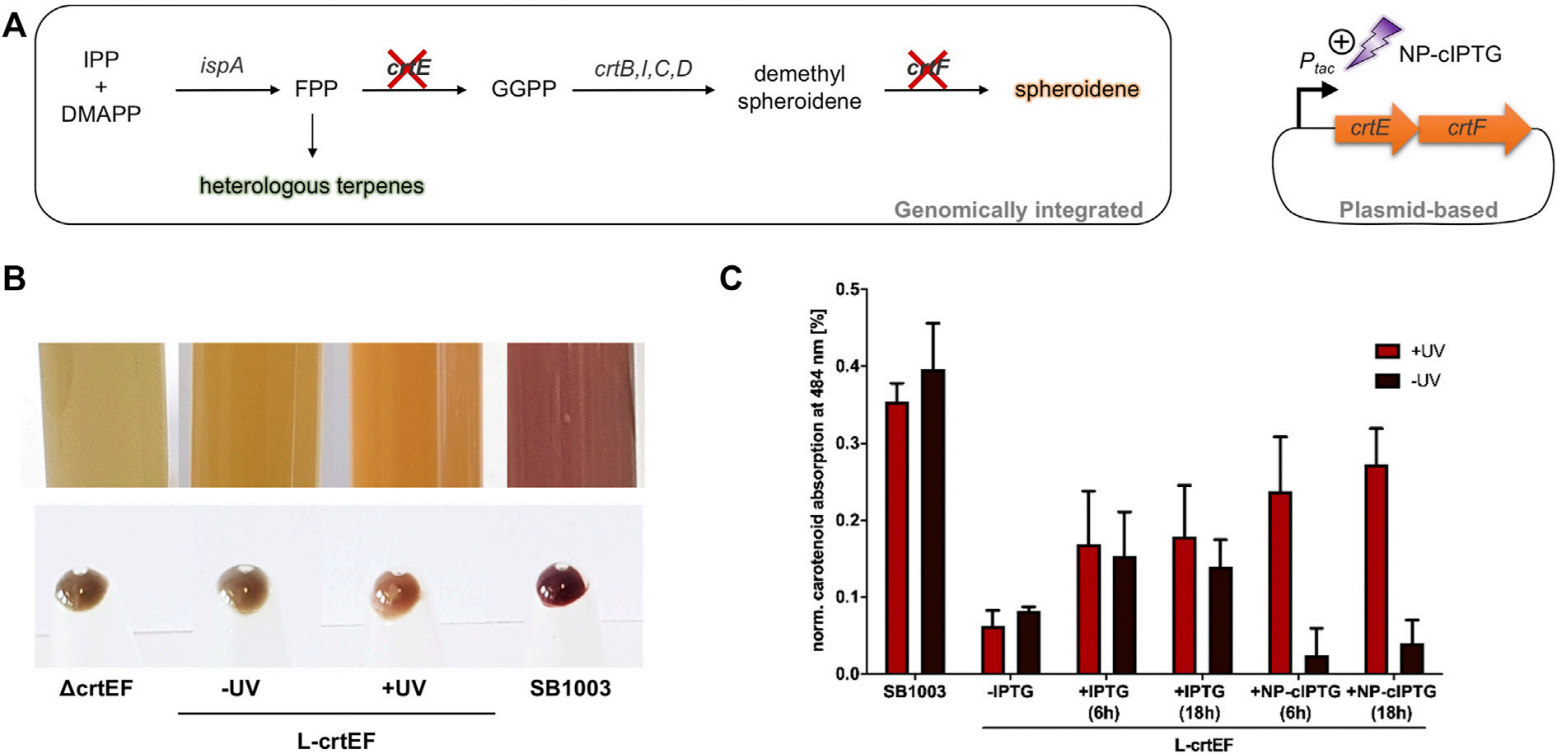
Light-controlled induction of carotenoid production in phototrophically grown *R. capsulatus* SB1003 *ΔcrtEF* cells carrying pRholtHi-crtE-crtF. **(A)** Intrinsic carotenoid synthesis of *R. capsulatus* starting from the terpene C_5_ building blocks IPP and DMAPP. Deletion of genes encoding the relevant carotenoid pathway enzymes CrtE and CrtF results in a carotenoid-deficient strain (Hage-Hülsmann et al., 2019). The genes *crtE* and *crtF* were cloned in a plasmid and placed under control of the P_*tac*_ promoter to facilitate IPTG-based control. Light-mediated induction of *crt* gene expression was achieved by using NP-cIPTG. IPP, isopentenyl pyrophosphate; DMAPP, dimethylallyl pyrophosphate; FPP, farnesyl pyrophosphate; IspA, FPP synthase; GGPP, geranylgeranyl pyrophosphate; CrtE, GGPP synthase; CrtB, phytoene synthase; CrtI, phytoene desaturase; CrtC, hydroxyneurosporene synthase; CrtD, hydroxyneurosporene desaturase; CrtF, demethylspheroidene O-methyltransferase. **(B)** Pigmentation of phototrophically grown *R. capsulatus* strains SB1003 *ΔcrtEF* (ΔcrtEF), SB1003 *ΔcrtEF*/pRholtHi-crtE-crtF supplemented with 1 mm NP-cIPTG under non-illuminated (-UV, L-crtEF) or UV-A illuminated conditions (+UV, L-crtEF) and SB1003 wildtype strain (SB1003). Besides liquid cultures, cell pellets corresponding to an optical density at 660 nm of 2.5 are shown. **(C)** Carotenoid absorption at 484 nm of *R. capsulatus* SB1003 *ΔcrtEF*/pRholtHi-crtE-crtF (L-crtEF) cultures supplemented with 1 mm NP-cIPTG (+NP-cIPTG + UV and -UV) are shown in comparison to the respective absorption of extracts from *R. capsulatus* SB1003 wildtype (SB1003) cells. In addition, cell cultures supplemented with 1 mm IPTG (positive control, +IPTG) or without IPTG (negative control, -IPTG) were likewise analyzed after 48 h of phototrophic cultivation [RCV medium, 30°C, screw neck vials, NIR light (*λ*_max_ = 850 nm, 1.7 mW/cm^2^)]. *R. capsulatus* SB1003 *ΔcrtEF* cultures were used to determine the background absorption at 484 nm and thus corresponding values were subtracted from all other values. Induction was performed after a cultivation time of six or 18 h *via* UV-A light exposure at 365 nm (∼2 mW/cm^2^) for 30 min. Values are means of individual biological triplicates. Error bars indicate the respective standard deviations.

As mentioned above, phototrophically grown *R. capsulatus* cultures exhibited a high basal *eyfp* expression level under uninduced conditions (Figure 3). Therefore, we next analyzed if the expression properties of the LacI/P_*tac*_ system can be further improved. In a recent publication, it was shown that the well-known shuttle vector pEKEx-2 (Eikmanns et al., 1991), which was used for isolating the *lacI* ^*q*^-P_*tac*_-lacO fragment in this work, showed likewise high basal target gene expression in uninduced *C. glutamicum* cultures (Bakkes et al., 2020). The authors proved that this plasmid features two unfavorable parts (Figure 4), which might hamper tight LacI-mediated gene expression and plasmid stability. Firstly, the nucleotide sequence of the repressor gene *lacI* revealed a deviation from the original *E. coli* K12 sequence at the 3′-end resulting in a substitution and elongation of the last 19 amino acid residues of LacI (starting from amino acid residue 341), which might lead to decreased repressor activity. Secondly, the plasmid backbone harbors a duplicated DNA sequence upstream of the *lacI* gene that corresponds to a correct LacI C-terminal coding sequence, which might lead to plasmid instability due to homologous recombination. After reconstitution of the original *lacI* sequence, leaky target gene expression could be significantly decreased (Bakkes et al., 2020). Following these recent findings, we replaced the likewise occurring wrong *lacI* gene variant on pRholHi-2-eYFP (in Figure 4 designated as L-eYFP) accordingly by an intact *lacI* and deleted the DNA duplicate region (519 bp) approximately 1.5 kb upstream of the *lacI* gene in the plasmid backbone (pRholHi-2_Δlac-eYFP; in Figure 5 designated as L_Δlac-eYFP). To analyze, if the *Rhodobacter* expression plasmid can be further improved, we finally implemented an *in silico* optimized ribosome binding site consisting of an optimized Shine-Dalgarno (SD) and SD-spacer sequence (pRholtHi-eYFP, in Figure 5 designated as Lt_Δlac-eYFP; for SD and spacer sequences, please see the Supplementary Material). Subsequently, LacI-controlled *eyfp* reporter gene expression in all three constructs was analyzed in *R. capsulatus* SB1003 under phototrophic conditions to evaluate the influence of those modifications on the basal expression level and induction factor of the respective expression systems (Figures 4B,C).

As already observed in the previous measurements, the expression plasmid pRholHi-2-eYFP leads to a high basal *eyfp* reporter gene expression with an induction factor of only ∼1.8 (Figures 4B,C). After reconstitution of the original *lacI* gene from *E. coli* and the deletion of the homologous *lacI* region in the plasmid backbone (pRholHi-2_Δlac-eYFP), the fluorescence level after induction was slightly decreased but the basal expression was also reduced leading to an induction factor of ∼2.5. However, the *in silico* calculation of an optimized RBS for *eyfp* expression in *R. capsulatus* (pRholtHi-eYFP) finally led to a 1.8-fold increased eYFP fluorescence level when compared to pRholHi-2-eYFP and an induction factor of ∼3.2. Further, the gradual inducibility of the novel plasmid variant pRholtHi-eYFP (Lt_Δlac-eYFP) was evaluated in *R. capsulatus* under both non-phototrophic and phototrophic growth conditions. For this purpose, IPTG was added to the medium at increasing concentrations (0–10 mm) after 9 h of cultivation (early logarithmic growth phase) and reporter gene expresion was analyzed by detecting the specific eYFP fluorescence (Supplementary Figures S2D–F). The optimized plasmid shows an improved induction profile with a gradual induction response. Additionally, the influence of the induction time point was evaluated under non-phototrophic and phototrophic growth conditions revealing the induction of reporter gene expression can be performed under both applied conditions in the lag, early, mid-logarithmic and late-logarithmic growth phase without having a strong influence on the growth or induction of eYFP gene expression (Supplementary Figure S3).

Further, toxicity measurements with the *R. capsulatus* SB1003 wildtype strain revealed no significant impairments of UV-A light or the photolysis products on cell growth (Supplementary Figure S6). Subsequently, this plasmid variant was used to reevaluate the cIPTG-mediated induction of *eyfp* expression in *R. capsulatus* under phototrophic conditions (Figure 4D). For this purpose, only NP-cIPTG was applied. As expected, the fluorescence intensity of cultures supplemented with IPTG and NP-cITPG after UV-A illumination was comparably high and the basal expression could be reduced (induction factor of ∼5.2) in comparison to the previous measurement (Figure 3A, induction factor for NP-cIPTG ∼2.9). Thus, not only the optimization of the plasmid backbone, but also the *in silico* optimization of the RBS sequence clearly increased the tightness and strength of this expression plasmid.

### Optochemical control of a secondary metabolite pathway in phototrophically grown *R. capsulatus*

Finally, we applied the light switch to modulate an intrinsic biosynthetic secondary metabolite pathway of *R. capsulatus*. We chose the early enzymatic step of the carotenoid pathway as suitable target, which is responsible for the conversion of the precursor molecule farnesyl pyrophosphate (FPP). FPP is a key metabolite that is essential for the synthesis of the photopigments spheroidene and spheroidenone (belonging to the class of tetraterpenoids) and heterologous sesqui- and triterpenoids (Figure 5A) (Troost et al., 2019; Hage-Hülsmann et al., 2021; Hilgers et al., 2021).

In a mutant strain lacking the relevant carotenoid biosynthesis genes *crtE* and *crtF*, intrinsic carotenoid biosynthesis cannot be performed, which results in a distinct greenish-colored phenotype (Figure 5B, Hage-Hülsmann et al., 2019). We used this strain for light-controlled complementation of the observed mutant phenotype, by placing *crtEF* expression under LacI/P_*tac*_ control. For this purpose, the plasmid pRholtHi-crtE-crtF was transferred to the carotenoid deletion strain *R. capsulatus* SB1003 *ΔcrtEF* and cells were grown phototrophically for 2 days under NIR light (*λ*_max_ = 850 nm, 1.7 mW/cm^2^). Firstly, the influence of the induction level on carotenoid production was evaluated by addition of IPTG concentrations ranging from 0 to 1 mm. Interestingly, in contrast to the eYFP reporter gene expression, increasing IPTG concentrations did not lead to gradually elevated carotenoid levels but rather to an on-or-off response possibly due to suboptimal fluxes of intermediates (Supplementary Figure S9). Nevertheless, this test system was subsequently evaluated for light-controlled regulation of carotenoid production using NP-cIPTG. Induction of the plasmid-encoded genes *crtE* and *crtF* was performed after six or 18 h *via* appropriate UV-A light exposure (30 min, *λ*_max_ = 365 nm, 2 mW/cm^2^) or by adding IPTG at the respective time point. For quantification of the carotenoid accumulation, the absorption of the cultures containing IPTG as well as *R. capsulatus* SB1003 wildtype and SB1003 *ΔcrtEF* cultures as corresponding positive and negative controls were analyzed using a Tecan Microplate reader (Figure 5C). While *R. capsulatus* SB1003 *ΔcrtEF* cultures without induction of *crtEF* expression exhibited a similar pigmentation as observed in the control strain (ΔcrtEF) (Figure 5B), addition of IPTG resulted in a clearly visible change of the cell coloration indicating a partial complementation of the phenotype that is caused by the *crtEF* gene deletion. This observation could be verified by quantitative analysis of the corresponding carotenoid absorption (Figure 5C). In addition, optochemical induction of *crtEF* gene expression using NP-cIPTG could almost completely restore the carotenoid-deficient phenotype resulting in a pronounced carotenoid absorption level thereby demonstrating the applicability of caged inducers as light-responsive on-switches for secondary metabolite biosynthesis under phototrophic conditions. This may open up new strategies for the dynamic control of complex secondary metabolite pathways such as the terpene pathway in phototrophic bacteria, which were recently shown to be promising alternative production hosts for the sustainable production of plant terpenes (Hage-Hülsmann et al., 2019; Troost et al., 2019; Hilgers et al., 2021; Klaus et al., 2022). In this context, the optochemical control of the carotenoid production might offer the possibility to precisely activate the carotenoid biosynthesis pathway in *R. capsulatus* cells allowing to modulate the FPP level for improving heterologous terpene production. This approach seems to be promising since an efficient terpene production requires a suitable FPP supply (Loeschcke et al., 2017; Troost et al., 2019; Hilgers et al., 2021). In near future, heterologous production of terpenes could thus be improved by performing phototrophic growth with optochemically controlled carotenoid levels to ensure an optimal supply of the FPP intermediate.

## Discussion

Two aspects were considered for establishing photocaged inducers as alternative optochemical on-switches suitable for light-driven induction of gene expression in the phototroph *R. capsulatus*: 1) the general functionality of photocaged inducers including their stability during cell cultivation, their non-toxicity, and their induction efficiency and 2) specific characteristics such as the compatibility of absorption spectra of photocaged inducers and photopigments as well as the robust applicability of the light-responsive inducer molecules under diverging growth conditions.

We could show that neither the three cIPTG derivatives nor the photoproducts are toxic for *R. capsulatus* and can basically be applied for inducing LacI/P_*tac*_-based target gene expression. However, only NP-cIPTG showed a sufficient stability in non-phototrophic and phototrophic *Rhodobacter* cultures when grown in the absence of UV-A light. The photoconversion of NP-cIPTG resulted in a specific activation of the P_*tac*_ promoter leading to a strong expression of downstream located genes irrespective of the chosen growth conditions. Remarkably, in some cases we could observe even higher induction levels when cIPTG instead of IPTG was used. These findings could be explained by an improved diffusion of the caged IPTG derivatives over the bacterial cell membrane in comparison to IPTG. Similar enhancement of photocaged inducer mediated induction of gene expression could also be observed in other bacterial expression hosts (Hogenkamp et al., 2021; Hogenkamp et al., 2022).

To retain the specific UV-A light responsiveness of cIPTG during phototrophic growth, we applied NIR light-emitting LED panels as alternative light sources. Irradiation of BChl *a* at 850 nm was shown to be sufficient for phototrophic growth thereby making the spectral range of 300–800 nm available for optochemical and optogenetic approaches. Besides the use of photocaged inducers these approaches thus may also include a variety of photoreceptors such as the well-known blue light-responsive photoreceptors of the LOV-, BLUF- or cryptochrome families (Briggs and Christie, 2002; Gomelsky and Klug, 2002; Lin and Todo, 2005; Losi and Gärtner, 2008; Drepper et al., 2011; Losi et al., 2018) or red light-sensing phytochromes (Levskaya et al., 2005; Rockwell et al., 2006; Tang et al., 2021).

Consequently, light-mediated regulation of gene expression in photosynthetic microbes using photocaged inducers opens up the possibility to optimize production of valuable secondary metabolites including terpenes. The combination of different optochemical and optogenetic switches further offers multi-factorial light-driven regulation approaches in principle allowing the gradual and dynamic control of complex biological processes with high spatial and temporal resolution. In future, this tool could thus prove valuable e.g., for automated control processes, where different light stimuli control cellular production processes in response to signals of intermediate- or product-specific biosensor in a closed-loop or microfluidic setup (Toettcher et al., 2011; Milias-Argeitis et al., 2016; Lalwani et al., 2018; Boada et al., 2020). Thus, photocaged inducers can contribute to non-invasive, light-mediated optimization of sustainable production processes in photosynthetic microbes.

## Material and methods

### Bacterial strains and plasmids

The *Escherichia coli* strain DH5α (Hanahan, 1983) was used for cloning and the strain S17-1 (Simon et al., 1983) for the conjugational transfer of expression plasmids. All *E. coli* strains were grown at 37°C on LB agar plates or in liquid LB medium (Luria/Miller, Carl Roth^®^), supplemented with kanamycin (50 μg mL^−1^) when necessary. The *R. capsulatus* strain SB1003 (Strnad et al., 2010) was grown on PY agar plates containing 2% Select Agar (Thermo Fisher Scientific) or in RCV liquid medium containing 15 mm ammonium and supplemented with kanamycin (25 μg mL^−1^) at 30°C. For cultivation of strain SB1003, rifampicin (25 μg mL^−1^) was used additionally. If not stated otherwise, cultivation was conducted under anaerobic, photoheterotrophic conditions and permanent illumination with bulb light (2500 lx), as described previously (Troost et al., 2019).

All bacterial strains as well as the construction and genetic properties of plasmids used in this study are listed in Supplementary Table S1.

### Cultivation of *R. capsulatus* for target gene expression

The aerobic and microaerobic expression cultures were grown in RCV + ammonium medium at 30°C using 48-well Round Well Plates^®^ in a BioLector microbioreactor system (m2p labs, Germany) applying variable filling volumes and shaking frequencies to control dissolved oxygen tension (800, 1,000 and 1,500 μL; 800 rpm or 400 rpm, respectively). Cultures were inoculated with an optical density of 0.1 determined at 660 nm. The cell density was measured during cultivation *via* scattered light intensity at 620 nm and the eYFP fluorescence intensities were online-monitored using a 508/532 nm filter. Heterologous and homologous gene expression was induced during early logarithmic phase (after approx. 9 h) *via* UV-A light exposure (VL-315. BL lamp, Vilber Lourmat, France; ∼1 mW/cm^2^, 30 min exposure) in RCV medium that was supplemented with BC-, BEC- or NP-cIPTG (1 mm) or by adding IPTG (1 mm) directly after light exposure in corresponding control cultures. In a previous study it could be demonstrated that these illumination conditions were sufficient for almost complete photoconversion of all cIPTG derivatives resulting in the full release of the active inducer molecule (Hogenkamp et al., 2021). The phototrophic expression cultures were inoculated with a starting OD_660nm_ of 0.5 in completely filled 4.2 mL screw neck vials (N13; Macherey-Nagel, Düren, Germany) using tight screw caps with a bonded septum (Macherey-Nagel, Düren, Germany) in RCV + ammonium medium at 30°C. Those were shown to be appropriate cultivation vessels for phototrophic growth in a previous study (Hilgers et al., 2021). To establish adequate environmental conditions for anoxygenic photosynthesis, cells were permanently illuminated with NIR light (850 nm, 1.7 mW/cm^2^) using customized LED panels that have been described previously (Hilgers et al., 2021). The expression was induced after 6 h by UV-A light irradiation (365 nm, 2 mW/cm^2^, 30 min exposure, the UV-A light LEDs are also installed on the panels) using NP-, BC-, or BEC-cIPTG (1 mm) added prior to cultivation. Due to the slightly higher distance of the vials to the LED panels during phototrophic cultivation in comparison to MTP and a possible shading effect of the increasingly colored *R. capsulatus* cells, we increased the UV-A light intensity to 2 mW/cm^2^ to ensure a full uncaging of cIPTG. The same concentration of IPTG was used for corresponding control experiments. After 48 h of cultivation, *R. capsulatus* biomass and eYFP fluorescence were determined. For comparable measurements, 800 µL of each vial were transferred to one well of a Round Well Plate and further incubated at 30°C for 1.5 h in a BioLector microbioreactor system (m2p labs, Germany) (800 μL, 800 rpm) to ensure O_2_-dependent eYFP chromophore maturation. After eYFP maturation was completed, the cell density was measured *via* scattered light intensity at 620 nm and the eYFP fluorescence was determined by using a 508/532 nm filter. It should be noted that the low solubility of NP-cIPTG in the cultivation medium can promote the formation of an emulsion-like solution, which can interfere with the determination of cell densities *via* the measurement of scattered light intensities in this experimental setup.

### DMNB-actinometry

For the DMNB assay, 1.25 mm DMNB was dissolved in aqueous KOH solution (0.5 M) with 10% (v/v) DMSO. The alkaline DMNB solution was subsequently incubated under the same conditions applied for phototrophic growth of *R. capsulatus*. DMNB photoconversion to MNP was monitored by the increase of absorption at a wavelength of 422 nm (100 μl, Tecan Infinite M1000 Pro microplate reader). The UV-A exposure was carried out using a UV-A hand lamp (VL-315. BL 45 W, Vilber Lourmat, France; 5.4 mW/cm^2^ at 365 nm for 1.5 cm distance to light source), the other light sources were used as described above.

### Carotenoid production

The carotenoid complementation assay was performed using 4.2 mL screw neck vials (N13; Macherey-Nagel, Düren, Germany) for cultivation of *R. capsulatus* SB1003 *ΔcrtEF* in NP-cIPTG supplemented RCV medium containing 15 mM ammonium [starting OD_660nm_ = 0.1, 30°C, anaerobic conditions, permanent illumination with NIR light (850 nm, 1.7 mW/cm^2^)]. The expression of the *crtEF* genes located on the P_*tac*_-based expression vector pRholtHi-crtE-crtF was induced after six or 18 h *via* UV-A light exposure (365 nm, 2 mW/cm^2^, 30 min exposure). IPTG was added at the same time points to the respective control strains. After 48 h, carotenoid accumulation was analyzed in 100 µL RCV *via* absorption measurement at 484 nm using an Infinite M1000 Pro microplate reader (Tecan Group LTD., Maennedorf, Switzerland).

For all illumination experiments, intensities of different light sources were quantified at relevant wavelengths (i.e., 365 and 850 nm) by using a Thermal Power Sensor (S302C, Thorlabs Inc., United States).

### Synthesis of photocaged compounds

NP-cIPTG (Young and Deiters, 2007; Binder et al., 2014), BEC-cIPTG and BC-cIPTG (Hogenkamp et al., 2021) were synthesized as published previously.

### Determination of compound purity by qNMR

The purity of the NP-cIPTG, BEC-cIPTG and BC-cIPTG was determined *via* quantitative NMR. (Methanesulfonyl)methane was utilized as internal standard for BC-cIPTG and 3,5-bis(trifluoromethyl)bromobenzene for NP-cIPTG as well as BEC-cIPTG. The spectra were measured at 20°C on a Bruker Avance/DRX 600 spectrometer with 64 scans each and 30 µs relaxation time between each scan. The results in Supplementary Table S2 are means of triplicate measurements.

## Data Availability

The original contributions presented in the study are included in the article/Supplementary Material, further inquiries can be directed to the corresponding authors.
