# Supplementary material for: Light-mediated control of gene expression in the anoxygenic phototrophic bacterium Rhodobacter capsulatus using photocaged inducers
Source: Front Bioeng Biotechnol. 2022 Sep 30;10:902059. doi: 10.3389/fbioe.2022.902059 (PMC9561348; doi:10.3389/fbioe.2022.902059)
Supplement: Supplementary file 1 [file DataSheet1.pdf]

Supplementary Material

Table of contents

1 Supplementary Methods **2**

Bacterial strains and plasmids 2

2 Supplementary Data **5**

Determination of suitable cultivation parameters for aerobic and microaerobic growth of *R. capsulatus* 5

Determination of IPTG concentrations sufficient for the induction of *eyfp* reporter gene expression in *R. capsulatus* under aerobic, microaerobic and phototrophic conditions 6

Influence of the induction time on the *eyfp* reporter gene expression and growth of *R. capsulatus* under microaerobic and phototrophic conditions 8

Toxicity and stability of photocaged IPTG variants in different cultivation media 10

Effect of UV-A light illumination on growth and *eyfp* reporter gene expression of *R. capsulatus* SB1003/pRholHi-2-eYFP cultures under aerobic and microaerobic conditions 12

Detailed toxicity analysis of UV-A light illumination and NP-cIPTG on growth of *R. capsulatus* SB1003 wildtype cultures under phototrophic conditions 13

DMNB-actinometry for photochemical monitoring of UV-A light exposure 14

Evaluation of NIR-light intensities for optimal phototrophic growth of *R. capsulatus* SB1003 expression cultures 15

Influence of increasing IPTG concentrations on the carotenoid production in NIR-illuminated *R. capsulatus* SB1003 *ΔcrtEF* cells harboring an expression plasmid carrying *crtEF* genes under P_{tac} control 16

DNA sequences of P_{tac} promoter regions including their RBS, RBS-spacers and/or MCS 17

DNA sequence of CrtE and CrtF from *R. capsulatus* SB1003 for cIPTG-mediated expression in *R. capsulatus* cultures 18

Determination of photocaged compound purity by qNMR 19

References 20

1 Supplementary Methods

Bacterial strains and plasmids

All bacterial strains, plasmids and oligonucleotides used in this study are listed in Table S1.

Table S1: Bacterial strains, plasmids and oligonucleotides used in this study.

Strains, plasmids, oligonucleotides	Relevant features, description or sequences	References
	**Strains**	
***E. coli* DH5α**	*F⁻ Φ80lacZΔM15 Δ(lacZYA-argF) U169 recA1 endA1 hsdR17 phoA supE44 thi-1 gyrA96 relA1 deoR*	[1]
***E. coli* S17-1**	Ec294::[RP4-2 (Tc^R::Mu)(Km^R::Tn7)] *recA, thi, pro, hsdR⁻ hsdM⁺* Tp^R, Sm^R	[2]
***E. coli* Tuner(DE3)**	*F⁻ompT hsdS*$_B$ *(*r_B^- m_B^-*) gal dcm lacY1*(DE3)	Novagen, Merck KGaA
***R. capsulatus* SB1003**	Wild-type, Rif^R	[3]
R. capsulatus* SB1003 *ΔcrtEF	*ΔcrtE, ΔcrtF*, Rif^R,Sm^R	[4]
	**Plasmids**	
**pRholHi-2-eYFP**	pBBR1-MCS-derivative, Km^R, Cm^R, with *Sma*I/*Xho*I inserted *lacI*^{q}-P$_{tac}$-lacO, *eyfp*	This work
**pRholHi-2_Δlac-eYFP**	pRholHi-2-eYFP derivative with homologous *lacI* fragment deletion and reconstituted *E. coli lacI*	This work
**pRholtHi-eYFP**	pRholHi-2-eYFP derivative with homologous *lacI* fragment deletion, reconstituted *E. coli lacI* and *in silico* calculated P$_{tac}$ RBS (i.e., Shine-Dalgarno and spacer sequence)	This work
**pRholtHi-crtE-crtF**	pBBR1-MCS-derivative, Km^R, Cm^R, with inserted *crtE crtF*	This work
**pRhofHi-2-eYFP**	pBBR1-MCS-derivative, Km^R, Cm^R, P$_{fru}$ with *Sma*I/*Xho*I inserted *eyfp*	Troost, unpublished
**pRhotHi-2-lacI-eYFP**	pBBR1-MCS-derivative, Km^R, Cm^R, pBBR22b-*lacI*, P$_{T7}$-lacO-MCS with *Nde*I *Xho*I inserted *eyfp*	[5]
	**Oligonucleotides**	
**1) SmaI_Ptac_eYFP_fw**	Binds at the 5’ end of the *lacI*$_q$ gene on pEKEX-2-eYFP plasmid, contains *Sma*I site. 5’-ATATCCCGGGCAAACATGGCCTGTCGC TTG-3’	This work
**2) XhoI_Ptac_eYFP_rev**	Binds at the 3’ end of the *eyfp* gene on pEKEX-e-eYFP plasmid, contains *Xho*I site. 5’-ATATCTCGAGCACACTACCATCGGCGC TAC-3’	This work
**3) pRholHi-dlacI _fw**	Binds at the 3`end of the plasmid pRholHi-2-eYFP downstream of the redundant *lacI* region, inserts homologous regions for InFusion® cloning. 5’-CGTTGCGCAATTCCGCGAACCCCAGAGT-3’	This work
**4) pRholHi-dlacI_rev**	Binds at the 5`end of the plasmid pRholHi-2-eYFP upstream of the redundant *lacI* region, inserts homologous regions for InFusion® cloning. 5’-CGGAATTGCGCAACGCAATTAATGTAAG TTAG-3’	This work

**5) pRholHi_fw**	Binds at the 3’ end of the plasmid pRholHi-2-eYFP downstream of the *lacI* gene variant.<br>5’-ATTCACCACCCTGAATTGACTCTC-3’	This work
**6) pRholHi_rev**	Binds at the 5’ end of the plasmid pRholHi-2-eYFP upstream of the *lacI* gene variant.<br>5’-GCGAGGGCGTGCAAGATTC-3’	This work
**7) lacI_EC_fw**	Binds at the 5’ end of the native *lacI* gene of *E. coli*, inserts homologous regions for InFusion® cloning.<br>5’-<u>CTTGCACGCCCTCGC</u>TCACTGCCCGCTTTC CAG-3’	This work
**8) lacI_EC_rev**	Binds at the 3’ end of the native *lacI* gene of *E. coli*, inserts homologous regions for InFusion® cloning.<br>5’-<u>TTCAGGGTGGTGAAT</u>GTGAAACCAGTAACG TTATACGATG-3’	This work
**9) pRholtHi_RBS_fw**	Binds at the 3’ end of the plasmid pRholHi-2_Δlac-eYFP and inserts homologous regions for InFusion® cloning.<br>5‘-<u>ACAAGGAGGTATTCA</u>TATGACCATGATT ACGCCAAGC-3‘	This work
**10) pRholtHi_RBS_rev**	Binds at the 5’ end of the plasmid pRholHi-2_Δlac-eYFP and inserts homologous regions for InFusion® cloning.<br>5‘-<u>TGAATACCTCCTTGT</u>GAAATTGTTATCCG CTCAC-3‘	This work
**11) CrtEF_genome_fw**	Binds at the 5’ end of the *crtE* gene in the *R. capsulatus* SB1003 genome, inserts homologous regions for InFusion® cloning.<br>5′-<u>AGGAGGTATTTTCAT</u>ATGTCTCTGG ATAAACGTATCGAG-3′	This work
**12) CrtEF_genome_rev**	Binds at the 3’ end of the *crtF* gene in the *R. capsulatus* SB1003 genome, inserts homologous regions for InFusion® cloning.<br>5’- <u>CTCGAATTCGGATCC</u>TCAGCCGC GTTCGGCCTC_-3’	This work
**13) pRholtHi-crtEF_fw**	Binds at the3’ end of the plasmid pRholtHi-eYFP.<br>5’- GGATCCGAATTCGAGCTCC-3’	This work
**14) pRholtHi-crtEF_rev**	Binds at the 5’ end of the plasmid pRholtHi-eYFP.<br>5’- ATGAAAATACCTCCTTGTGAAATTG -3’	This work
**15) pRholtHi-crtEF-QC_fw**	Binds at the RBS of plasmid pRholtHi-crtE-crtF and inserts optimized RBS/spacer<br>5’- CAAGGAGGTTTCATATGTCTCTG GATAAACG -3’	This work
**16) pRholtHi-crtEF-QC_rev**	Binds at the RBS of plasmid pRholtHi-crtE-crtF and inserts optimized RBS/spacer<br>5’- TATGAAACCTCCTTGTGAAA TTGTTATCCG -3’	This work

All recombinant DNA techniques were carried out using *E. coli* DH5α as described by Sambrook *et al.*[6]. Construction of expression vectors were carried out using restriction and ligation cloning. The PCR fragment containing the gene for lacI^{q} and the P$_{tac}$ promoter was amplified from a synthetic gene construct on the shuttle vector pEKEx-2 (Eurofins Genomics, Ebersberg, Germany) with oligos 1 and 2 generating appropriate *Sma*I/*Xho*I restriction sites at

its 5'- and 3'-ends. This fragment was inserted into the likewise hydrolyzed plasmid pRhofHi-2-eYFP to build the plasmid pRholHi-2-eYFP.
For construction of the optimized pRholHi-2_Δlac-eYFP plasmid, the redundant 1.5 kb *lacI* fragment upstream of the original *lacI* gene was deleted and the *lacI* gene variant was replaced by the original *lacI* gene from *E. coli* K12. Both changes were conducted in one cloning step. For this purpose, three PCR fragments were generated: the first one encompasses the region between the deleted redundant *lacI* fragment and the original *lacI* gene of the plasmid backbone (oligos 3 and 6), the second one encompasses a native version of the *lacI* gene from *E. coli* (oligos 7 and 8) and the third one encompasses the plasmid backbone after the *lacI* gene until the redundant *lacI* region (oligos 4 and 5). Subsequently, the three fragments were assembled *via* InFusion® Cloning (Takara Bio Europe, St Germain en Laye, France).
The plasmid pRholtHi-eYFP was constructed using the pRholHi_Δlac-eYFP by changing the RBS consisting of the Shine-Dalgarno and spacer sequence upstream of the *eyfp* reporter gene. An *in silico* optimized RBS and RBS-spacer including an NdeI site prior to the start codon of *eyfp* (for sequences see chapter "DNA sequences of P_{tac} promoter regions including their RBS, RBS-spacers and/or MCS") was calculated with the Salis Lab RBS calculator (https://salislab.net/software/predict_rbs_calculator) [7]. The plasmid backbone was amplified *via* PCR using olios 9 and 10, which featured homologous regions containing the optimized RBS and reassembled *via* InFusion® Cloning (Takara Bio Europe, St Germain en Laye, France).
For the plasmid pRholtHi-crtE-crtF, the genes *crtE* and *crFE* were amplified from the genome of *R. capsulatus* SB1003 with oligos 11 and 12 generating appropriate homologous regions for InFusion® Cloning (Takara Bio Europe, St Germain en Laye, France) at their 5'- and 3'-ends. The plasmid backbone was amplified *via* PCR from the pRholtHi-eYFP using oligos 13 and 14. The PCR fragment was inserted into the amplified backbone to build the plasmid pRholtHi-crtE-crtF. Additionally, an *in silico* optimized RBS and RBS-spacer including an NdeI site prior to the start codon of *crtE* (for sequences see chapter "DNA sequence of CrtE and CrtF from *R. capsulatus* SB1003 for cIPTG-mediated expression in *R. capsulatus* cultures") was calculated with the Salis Lab RBS calculator (https://salislab.net/software/predict_rbs_calculator) [7]. The adaption of the RBS spacer was performed *via* QuikChange using oligos 15 and 16.

2 Supplementary Data

Determination of suitable cultivation parameters for aerobic and microaerobic growth of *R. capsulatus*

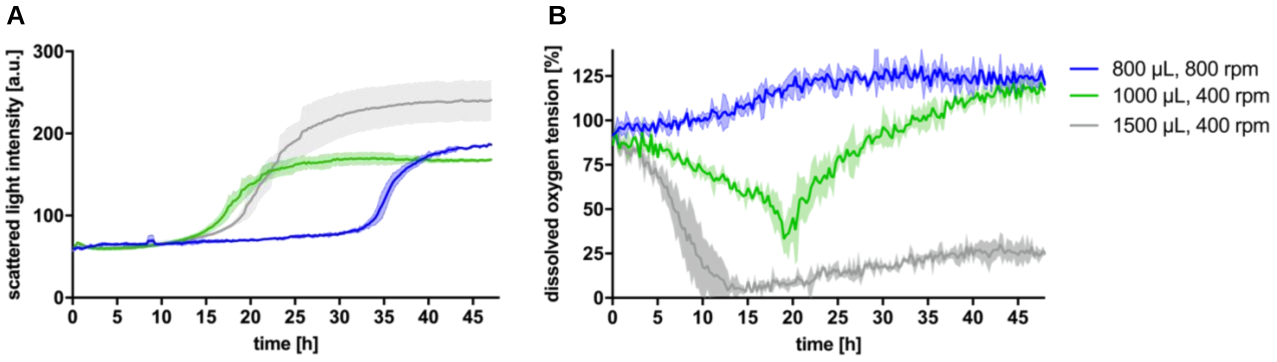

Figure S1: (A) Cell growth of *R. capsulatus* SB1003/pRholHi-2-eYFP expression cultures under varying filling volumes and shaking frequencies. The bacteria were grown in RCV medium for 48 h in Round Well Plates in the dark at 30 °C. To identify filling volumes and shaking frequencies that are appropriate for aerobic and microaerobic growth, the following cultivation conditions were applied: (i) 800 rpm and with 800 µL RCV medium (blue), (ii) 400 rpm and 1000 µL RCV medium (green) and (iii) 400 rpm and 1500 µL RCV medium (grey). Cell growth was analyzed by determining the scattered light intensity using a BioLector system. Values are means of individual biological triplicates. Error bars indicate the respective standard deviations. **(B)** Dissolved oxygen tension (DOT) of the same *R. capsulatus* cultures as described in A). The DOT was determined in plates with oxygen sensitive optodes during cultivation of *Rhodobacter* in the BioLector system (λ_{ex} = 520 nm, λ_{em} = 600 nm). Values are means of individual biological triplicates. Error bars indicate the respective standard deviations.

To control the oxygen level during non-phototrophic growth, the filling volume of the Round Well plate as well as the shaking frequencies were appropriately adapted so that a maximal aeration (around 100%) and a minimal aeration (< 25%) were maintained throughout the cultivation. For this purpose, bacterial growth (Figure S1A) and the dissolved oxygen tension (DOT; Figure S1B) were online-monitored using the scattered light intensity and DO optodes (m2p-labs, Germany), respectively. A filling volume of 800 µL and a shaking frequency of 800 rpm resulted in a constant oxygen tension of 100% (Figure S1B, blue line). To reduce the DOT during *R. capsulatus* cultivation, a filling volume of 1000 µL and a shaking frequency of 400 rpm were applied. However, these conditions were not sufficient for constant microaerobic growth, as the oxygen tension increased up to 100% during stationary growth phase (Figure S1B, green line). Therefore, a lager filling volume (1500 µL) at the same shaking frequency were used, which led to a strongly decreased oxygen tension of under 25% during both logarithmic and stationary growth phase (Figure S1B, grey line).

Determination of IPTG concentrations sufficient for the induction of *eyfp* reporter gene expression in *R. capsulatus* under aerobic, microaerobic and phototrophic conditions

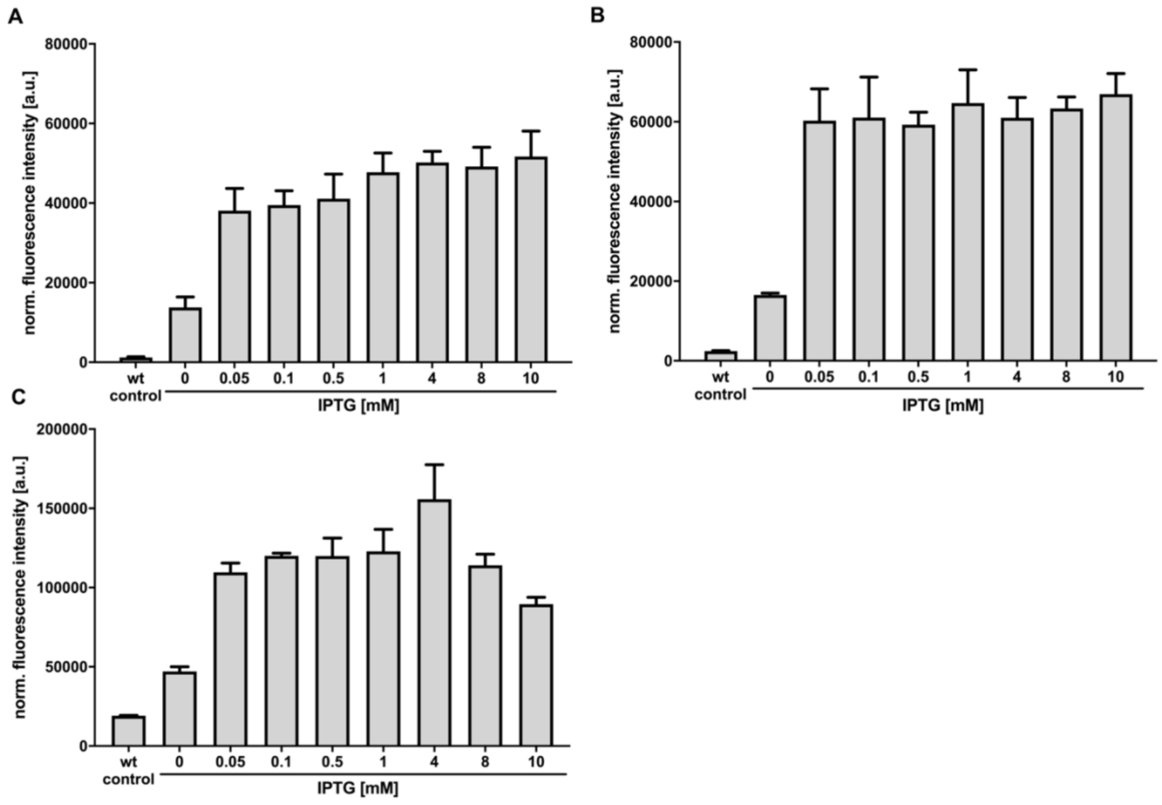

Figure S2: Relative eYFP fluorescence intensities of *R. capsulatus* SB1003/pRholHi-2-eYFP cultures grown aerobically **(A)**, microaerobically **(B)** or anaerobically **(C)** and supplemented with increasing amounts of IPTG. Aerobic and microaerobic cultures were incubated in 800 µL or 1500 µL RCV medium and a shaking frequency of 800 rpm or 400 rpm for 48 h in the dark at 30 °C. Induction of gene expression was performed after 9 h by adding IPTG at concentrations ranging from 0 to 10 mM. Phototrophic cultures were incubated in 4.2 mL RCV medium for 48 h at 30 °C under constant illumination with bulb light (2000 lx) and IPTG was added prior to culture inoculation. *In vivo* fluorescence intensities of all cultures were determined by using a BioLector system (eYFP: λ_{ex} = 508 nm, λ_{em} = 532 nm) and values were normalized to cell densities. Values are means of individual biological triplicates. Error bars indicate the respective standard deviations.

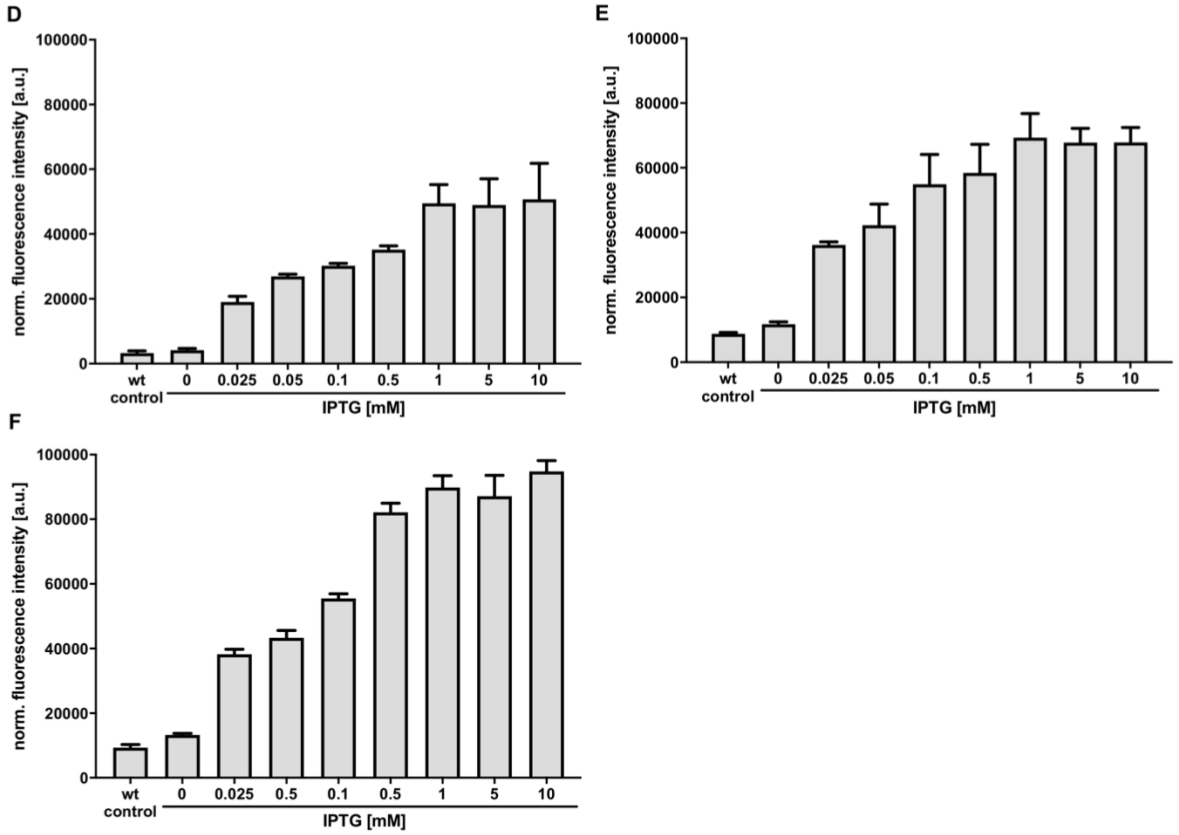

Continued figure S2: Relative eYFP fluorescence intensities of *R. capsulatus* SB1003/pRholtHi-eYFP cultures grown aerobically **(D)**, microaerobically **(E)** or anaerobically **(F)** and supplemented with increasing amounts of IPTG. Aerobic and microaerobic cultures were incubated in 800 µL or 1500 µL RCV medium and a shaking frequency of 800 rpm or 400 rpm for 48 h in the dark at 30 °C. Induction of gene expression was performed after 9 h by adding IPTG at concentrations ranging from 0 to 10 mM. Phototrophic cultures were incubated in 4.2 mL RCV medium for 48 h at 30 °C under constant illumination with bulb light (2000 lx) and IPTG was added prior to culture inoculation. *In vivo* fluorescence intensities of all cultures were determined by using a BioLector system or a Tecan Microplate reader (eYFP: λ_{ex} = 508 nm, λ_{em} = 532 nm) and values were normalized to cell densities. Values are means of individual biological triplicates. Error bars indicate the respective standard deviations.

Considerably high *eyfp* expression levels could be seen for IPTG concentrations of 1 mM and above, thus 1 mM was chosen as a sufficient inducer concentration in all further experiments. The induction profile using the optimized expression plasmid pRholtHi-eYFP shows an enhanced gradual induction in comparison to the precedent plasmid.

Influence of the induction time on the *eyfp* reporter gene expression and growth of *R. capsulatus* under microaerobic and phototrophic conditions

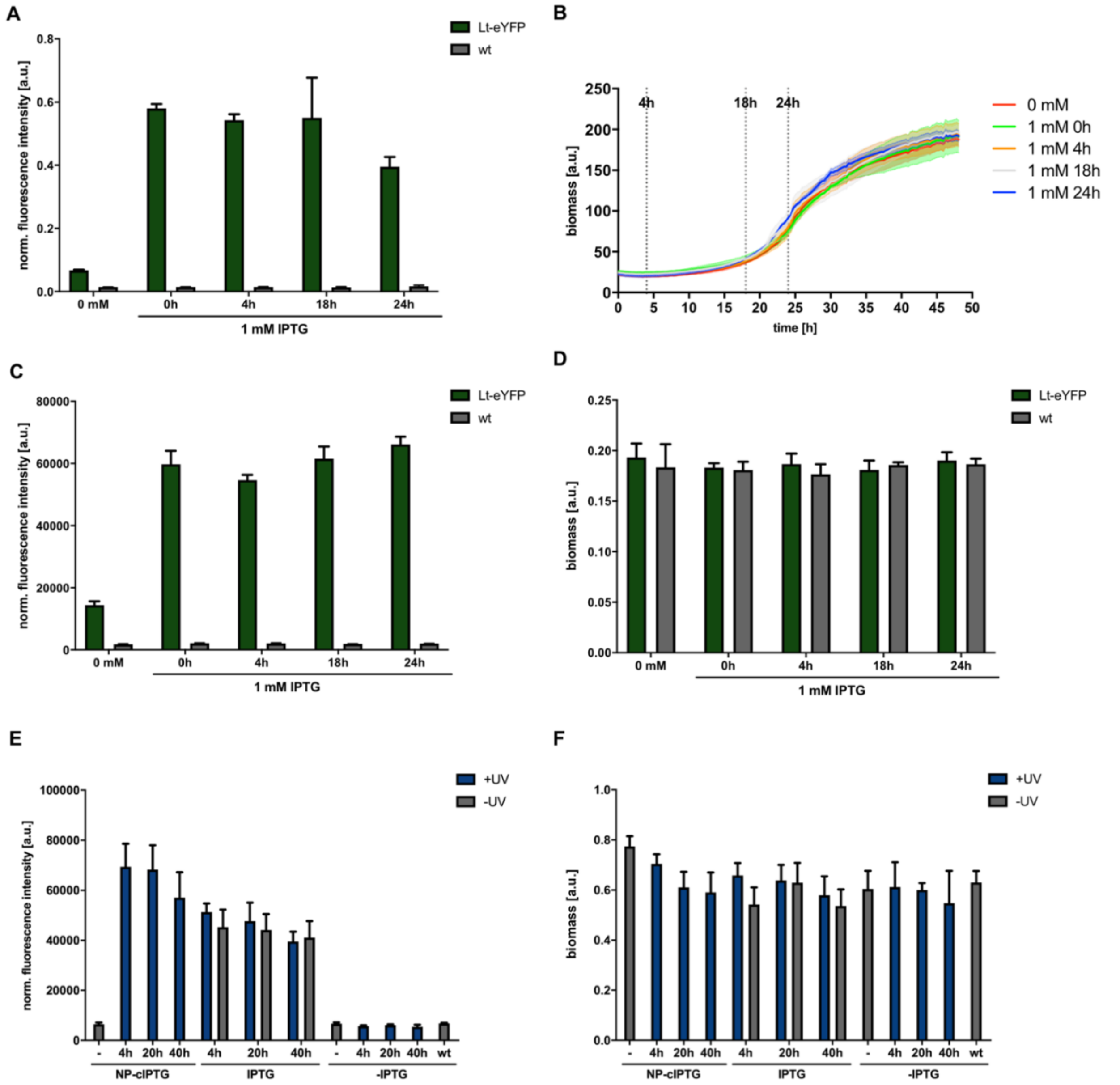

Figure S3: Effect of different induction time-points on eYFP fluorescence intensities and cell growth of *R. capsulatus* SB1003/pRholtHi-eYFP cultures grown microaerobically **(A,B)** or anaerobically **(C,D).** For induction of eYFP gene expression, IPTG was supplemented at different time points (0h, 4h = lag phase, 18 h = early logarithmic growth phase, and 24h = mid-logarithmic growth phase). Microaerobic cultures were incubated in 1500 µL RCV medium and a shaking frequency of 400 rpm for 48 h in the dark at 30 °C. Phototrophic cultures were incubated in 4.2 mL RCV medium for 48 h at 30 °C under constant illumination with bulb light (2000 lx). Additionally, the influence of increasing cell densities on NP-cIPTG uncaging was evaluated in anaerobically grown *R. capsulatus* cultures **(E,F)**. For light-induction of eYFP gene expression, growth media were supplemented with 1 mM NP-cIPTG prior cultivation and cultures were subsequently illuminated with UV-A light (365 nm, ~2 mW/cm^2) for 30 min at the given time points (4h = lag phase, 20 h = early logarithmic growth phase, and 40h = late logarithmic growth phase) or kept in the dark (-). Corresponding control cultures were supplemented with 1mM IPTG at the same time points. While cultures that were induced after both 4h and 20h have 44h for eYFP production (fluorescence measurement after 48h or 64h of the total cultivation time, respectively), the cultivation time could not be expanded accordingly

for cultures induced after 40h, because *R. capsulatus* cultures start to die after approximately 72h under the here applied cultivation conditions. For those cultures, the fluorescence measurement was conducted after 72h of the total cultivation time, corresponding to an eYFP production time of 32h. *In vivo* fluorescence intensities (eYFP: λ_{ex} = 508 nm, λ_{em} = 532 nm) and biomass of all cultures (scattered light intensity at 620 nm or absorption at 660 nm, respectively) were determined at the above stated time points by using a BioLector system or a Tecan Microplate reader and fluorescence values were normalized to cell densities. Values are means of individual biological triplicates. Error bars indicate the respective standard deviations.

The results demonstrate that the induction and uncaging works well even under higher cell densities. The slightly lower fluorescence intensity after 40h in **Fig. S5 E** is most probably due to a shorter time span from induction to the final eYFP measurement.

Toxicity and stability of photocaged IPTG variants in different cultivation media

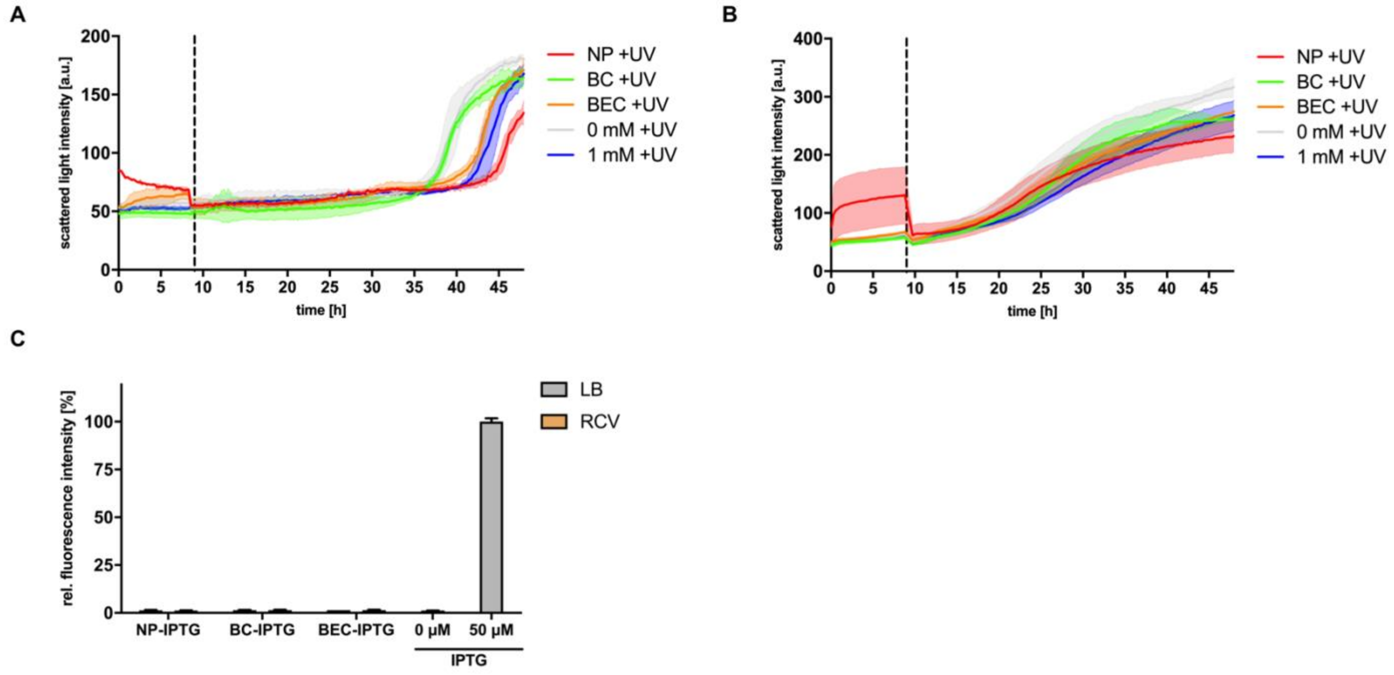

Figure S4: A-B) Growth curves of *R. capsulatus* SB1003/pRholHi-2-eYFP expression cultures in the presence of the photocaged IPTG variants compared to an uninduced (0 mM) and induced (1 mM IPTG) expression culture. Cells were grown over 48 h using a BioLector system (RCV medium supplemented with 1 mM of each caged compound, 30°C, 800 rpm and 800 µL filling volume for aerobic cultures **(A)**, 400 rpm and 1500 µL for microaerobic cultures **(B)**). Cell growth was analyzed by determining the scattered light intensity. After 9 h, formation of photoproducts was induced in cultures of *R. capsulatus via* light exposure at 365 nm (~1 mW/cm^2, indicated by dashed lines) for 30 min. The addition of IPTG was used as a control to comparatively analyze cell growth. Values are means of individual biological triplicates. Error bars indicate the respective standard deviations. **C)** eYFP *in vivo* fluorescence intensity of *E. coli* Tuner (DE3)/pRhotHi-2-lacI-eYFP expression cultures supplemented with the photocaged inducers which were previously incubated under different conditions. The three caged inducers (1 mM) were either incubated in LB (grey bars) or RCV (orange bars) medium for 48 h at 30 °C and added to the *E. coli* cultures afterwards in appropriate concentrations resulting in an end concentration of 50 µM. The *E. coli* cultures were incubated in the dark for 20 h in LB medium at 30 °C. *In vivo* stability of the photocaged IPTG variants is reflected by a low induction of reporter gene expression. As positive control, *E. coli* Tuner (DE3)/ pRhotHi-2-lacI-eYFP cultures supplemented with and without IPTG were used. *In vivo* fluorescence intensities were determined by using a BioLector system (eYFP: λ_{ex} = 508 nm, λ_{em} = 532 nm) and are shown in relation to the respective fluorescence intensities of the positive control cultures (50 µM IPTG). All values are normalized to cell-densities and are means of individual biological triplicates. Error bars indicate the respective standard deviations.

The elevated scattered light values at the beginning of the cultivation (i.e., before UV-A light exposure, Figure S4 A and B) can be attributed to the poorer water solubility of NP-cIPTG at concentrations of 1 mM, as a certain amount of these compounds initially form emulsions in the cultivation medium. Consequently, they contribute significantly more to the scattered light value than the bacterial cells that are initially still present in low numbers. However, exposure

to UV-A light dissolves these emulsions, which is reflected by the rapid decrease in the scattered light intensity after 9 h.

This control experiment with the well-established expression strain *E. coli* Tuner(DE3) [5] indicated that cIPTG instability was not detectable in sole LB or RCV medium. This gives a first hint that the instability of BC-cIPTG and BEC-cIPTG might be caused by using *R. capsulatus* as expression host, probably due to host specific enzymes or metabolism products.

Effect of UV-A light illumination on growth and *eyfp* reporter gene expression of *R. capsulatus* SB1003/pRholHi-2-eYFP cultures under aerobic and microaerobic conditions

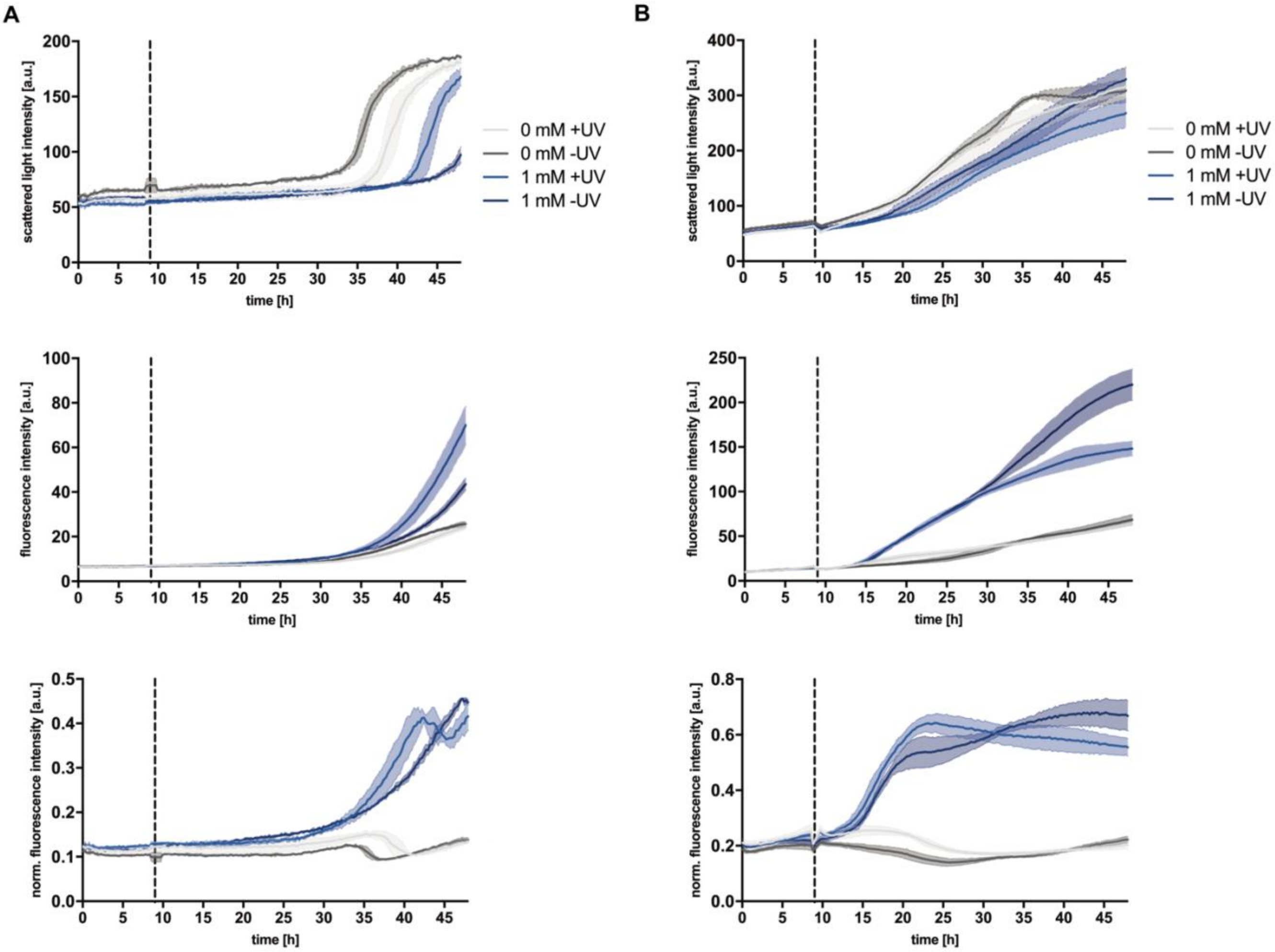

Figure S5: Cell growth and eYFP fluorescence during cultivation of *R. capsulatus* SB1003/pRholHi-2-eYFP. Cells were grown over 48 h in RCV medium at 30°C, 800 rpm and with 800 µL filling volume for aerobic conditions **(panel A)** and 400 rpm and 1500 µL for microaerobic conditions **(panel B)** using a BioLector system. Induction of eYFP gene expression was performed using 1 mM of IPTG (blue lines; uninduced grey lines). Simultaneously, cultures were incubated with (light lines) and without (dark line) UV-A light exposure at 365 nm (~1 mW/cm^2) for 30 min. The time point of induction is indicated by the dashed lines (9 h). Cell growth was analyzed by determining the scattered light intensity and the *in vivo* eYFP fluorescence intensities were measured at λ_{ex} = 508 nm, λ_{em} = 532 nm, as depicted in both center graphs in the middle, and normalized to cell densities, as shown in the bottom graphs. Values are means of individual biological triplicates. Error bars indicate the respective standard deviations.

Detailed toxicity analysis of UV-A light illumination and NP-cIPTG on growth of *R. capsulatus* SB1003 wildtype cultures under phototrophic conditions

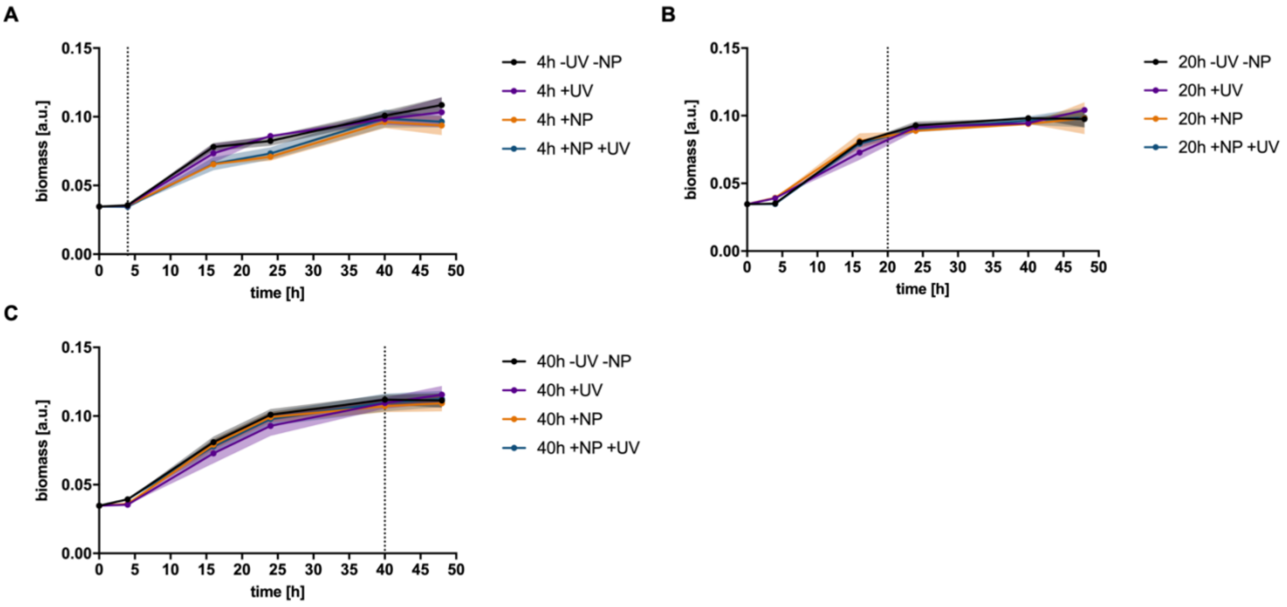

Figure S6: Cell growth during phototrophic cultivation of *R. capsulatus* SB1003 wildtype. Cells were grown over 48 h in RCV medium at 30°C in screw neck vials under NIR light (λ_{max} = 850 nm, 1.7 mW/cm^2). At the indicated time points, cultures were supplemented with photolysis product (NP) resulting in an end concentration of 1 mM (orange lines), illuminated with UV-A light at 365 nm (~2 mW/cm^2) for 30 min (purple lines). The photolysis product was generated by a complete uncaging of an NP-cIPTG solution under UV-A light. Simultaneously, cultures were incubated in the presence of UV-A light and photolysis products (blue lines). The time point of illumination and/or addition of NP-cIPTG photolysis products (4h, 20h, or 40h) are indicated by the dashed lines. *R. capsulatus* cells that have been cultivated phototrophically without UV-A exposure and addition of NP-cIPTG photoproducts were used as a corresponding control experiment (black lines). Cell growth was analyzed by determining the absorption at 660 nm using a Tecan Microplate reader. Values are means of individual biological triplicates. Error bars indicate the respective standard deviations.

DMNB-actinometry for photochemical monitoring of UV-A light exposure

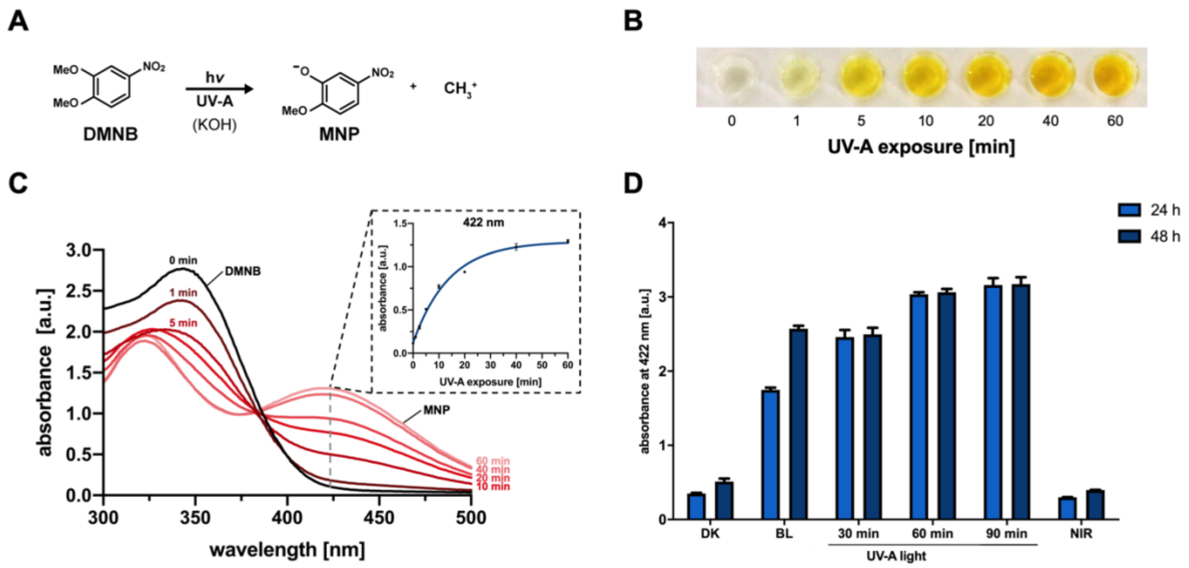

Figure S7: Photochemical monitoring of UV-A light exposure using DMNB actinometry. **(A)** Photochemical formation of 2-methoxy-5-nitrophenolate (MNP) from 1,2-dimethoxy-4-nitrobenzene (DMNB) using UV-A light in aqueous potassium hydroxide solution [8]. **(B)** Colorimetric changes of DMNB solution upon increased UV-A exposure. **(C)** Light-mediated conversion of DMNB (1.25 mM) after 0, 1, 5, 10, 20, 40 and 60 min of mid-power UV-A light exposure (5.4 mW/cm^2). The grey dashed line indicates respective maximal absorption differences of ΔA_{max}= 422 nm in the blue range; the insert shows the relation between absorbance at 422 nm and the duration of UV-A exposure. **(D)** Light-mediated formation of 2-methoxy-5-nitrophenolate (MNP) from a 1.25 mM DMNB solution in aqueous potassium hydroxide was determined spectrophotometrically *via* the increase of absorbance at 422 nm after illumination with bulb light (BL, 2500 lx) and NIR light (NIR, λ_{max} = 850 nm; 1.23 mW/cm^2) for 24 h or 48 h. DMNB photoconversion was compared to samples that have been exposed to UV-A light for 30–90 min (λ_{max} = 365 nm; 5.4 mW/cm^2) or kept unexposed (dark control, DK). Values are means of triplicate measurements. Error bars indicate the respective standard deviations.

Evaluation of NIR-light intensities for optimal phototrophic growth of *R. capsulatus* SB1003 expression cultures

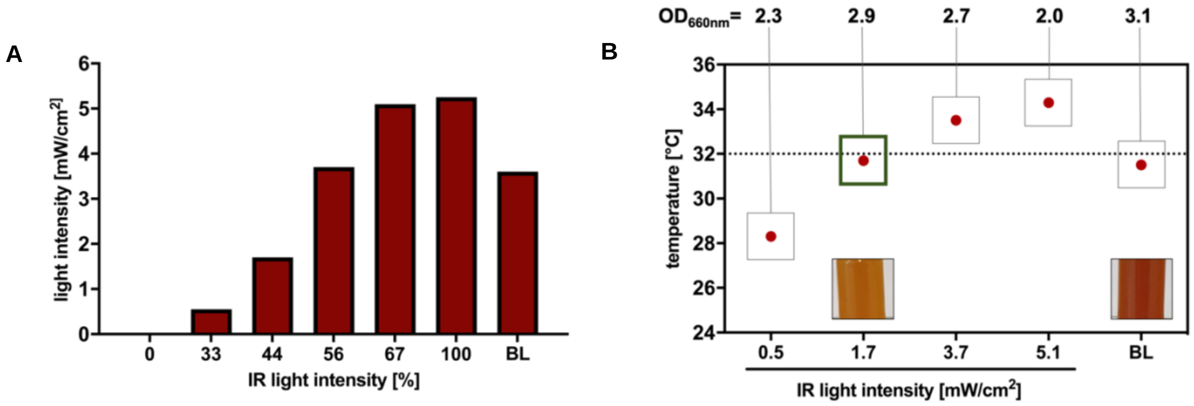

Figure S8: (**A**) Detected NIR light intensities [mW/cm^2] at λ_{max}= 850 nm of NIR panels from Vossloh-Schwabe [9] for increasing intensity settings, which can be set *via* a rotary knob with a continuous adjustment are shown in comparison to the NIR light amount of bulb light (BL) at λ_{max}= 850 nm. Light intensity quantifications were conducted using a Thermal Power Sensor (S302C, Thorlabs Inc, USA). (**B**) Medium temperature at suitable NIR light intensities after 48 h of cultivation and two pictures of corresponding *R. capsulatus* SB1003 cultures in the cultivation vessel as well as the respective optical density at 660 nm. The cultivation temperature of 33 °C should not be exceeded to avoid adverse effects on cell growth. The culture without any growth impairment and with the highest cell density is marked with a green frame and can be compared to cultures exposed with bulb light (BL).

For the determination of suitable NIR light intensities for efficient phototrophic growth of *R. capsulatus*, cells were cultivated with NIR light of increasing intensities ranging from 0.5 mW/cm^2 up to 5.1 mW/cm^2 (Figure S5 A) and analyzed with respect to their growth behavior (Figure S5 B). Screw neck vials, which were used as cultivation vessels, were placed in a distance of approx. 10 cm from each NIR panel. Adequately grown cultures without any sunken cells could only be detected for NIR light intensities of 1.7 mW/cm^2 and 3.1 mW/cm^2 represented by cell numbers corresponding to an optical density at 660 nm of 2.9 and 2.7, which are comparable to the optical density of cultures grown in bulb light (OD_{660nm}= 3.1). Lower or higher NIR light intensities led to decreased cell densities and unequally distributed cultures with sunken cells indicating a hampered cell viability. Presumably, this was on the one hand due to the insufficient exposure intensity and on the other hand due to the excessively high temperature of over 33 °C in the cultivation medium for the highest NIR light intensity. For all following experiments, an NIR light intensity of 1.7 mW/cm^2 was chosen as this condition offers an appropriate medium temperature and the highest cell density after 48 h.

Influence of increasing IPTG concentrations on the carotenoid production in NIR-illuminated *R. capsulatus* SB1003 *ΔcrtEF* cells harboring an expression plasmid carrying *crtEF* genes under P_{tac} control

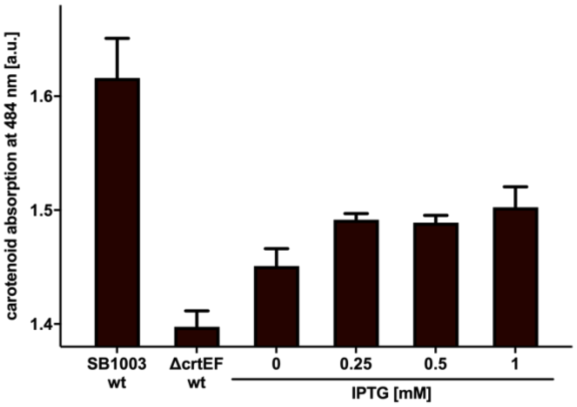

Figure S9: Carotenoid absorption at 484 nm of *R. capsulatus* SB1003 *ΔcrtEF*/ pRholtHi-crtE-crtF (L-crtEF) supplemented with IPTG concentrations ranging from 0 to 1 mM are shown in comparison to the respective absorption of *R. capsulatus* SB1003 wildtype (SB1003 wt) cell extracts. In addition, *R. capsulatus* SB1003 *ΔcrtEF* (*ΔcrtEF* wt) cultures were likewise analyzed after 48 h of phototrophic cultivation (RCV medium, 30 °C, screw neck vials, NIR light (λ_{max} = 850 nm, 1.7 mW/cm^2)). Induction was performed after a cultivation time of 6 h *via* addition of IPTG. Carotenoid absorption values are normalized to the cell densities. Values are means of individual biological triplicates. Error bars indicate the respective standard deviations.

DNA sequences of P$_{tac}$ promoter regions including their RBS, RBS-spacers and/or MCS

pEKEx-2:

P$_{tac}$ promoter **SD** **lac operator**

TTGACAATTAATCATCGGCTCGTATAATGTGTGGAA**TTGTGAGCGGATAACA**
ATTTCACACAGGAAACAGAATTAAAAGATATGACCATGATTACGCC**AAGCTTGC**
ATGCCTGCAGGTCGACTCTAGAGGATCCCCGGGTACCGAGCTCGAATTC

pUC18/19 MCS **BamHI**

pRholHi-2-eYFP and pRholHi-2_Δlac-eYFP:

P$_{tac}$ promoter **SD** **lac operator**

TTGACAATTAATCATCGGCTCGTATAATGTGTGGAA**TTGTGAGCGGATAACA**
ATTTCACACAGGAAACAGAATTAAAAGAT**ATG**ACC**ATG**ATTACGCC**AAGCTTGC**
ATGCCTGCAGGTCGACTCTAGAGGATCCA**AGGAG**ATATAGAT**ATG**-***eyfp***

pUC18/19 MCS **BamHI** **optimized SD**

8 bp RBS-spacer (between Shine-Dalgarno sequence and start codon)

pRholtHi-eYFP:

P$_{tac}$ promoter **lac operator**

TTGACAATTAATCATCGGCTCGTATAATGTGTGGAA**TTGTGAGCGGATAACA**
ATTTCACA**AGGAG**GTATTCAT**ATG**-***eyfp***

optimized SD **NdeI**

Optimized 8 bp RBS-spacer (between Shine-Dalgarno sequence and start codon)

DNA sequence of CrtE and CrtF from *R. capsulatus* SB1003 for cIPTG-mediated expression in *R. capsulatus* cultures

The shown sequence encompasses the *crtE* (red bases) and *crtF* gene (blue bases) referring to the mRNA sequence published in GenBank: CP001312.1, encoding the GGPP synthase CrtE and the demethylspheroidene O-methyltransferase CrtF (UniProtKB: P17060 and P17061, respectively). The sequence was optimized using a 6 bp RBS-spacer between the Shine-Dalgarno sequence and start codon.

[…]lacO **RBS** **opt. SD** **Start** *crtE*
<u>TAACAA</u>TTTCAC**AAGGAGG****TTTCAT****ATG**TCTCTGGATAAACGTATCGAGTCGGC
GCTGGTCAAGGCGCTGTCACCCGAGGCTTTGGGTGAATCTCCGCCGTTGCTTGCC
GCCGCGCTGCCTTACGGGGTGTTTCCCGGCGGCGCGGATCCGGCCGACGATCC
TTGTCTCGGTCGCGCTCGCCTGTGGCGACGATTGCCCGGCGGTCACCGATGCCGC
GGCCGTGGCGCTGGAGCTGATGCATTGCGCGAGCCTCGTGCATGACGATCTGCCC
GCCTTCGACAATGCCGACATCCGGCGCGGCAAGCCGAGCCTTCACAAGGCCTATA
ATGAACCGCTTGCGGTTCTGGCGGGCGACAGCCTGCTGATCCGCGGCTTCGAAGT
GCTGGCCGATGTCGGCGCCGTCAACCCGGACCGGGCGCTGAAGCTGATCTCGAA
ACTGGGTCAGCTGTCGGGGGCGCGCGGCGGGATCTGCGCCGGTCAGGCCTGGGA
AAGCGAATCCAAGGTCGATCTGGCCGCCTATCATCAGGCGAAGACCGGGGCGCT
GTTCATTGCCGCGACCCAGATGGGGGCGATTGCGGCGGGCTACGAGGCCGAACC
CTGGTTCGATCTGGGCATGCGGATCGGCTCGGCCTTCCAGATCGCCGACGACCTG
AAAGACGCGCTGATGTCGGCCGAGGCAATGGGCAAGCCCGCCGGGCAGGACATC
GCGAACGAACGCCCGAATGCGGTCAAGACGATGGGCATCGAGGGCGCGCGCAAA
CATCTGCAAGATGTGCTGGCGGGGGCGATCGCCTCGATCCCGTCCTGCCCCGGTG
AGGCGAAGCTGGCCCAGATGGTGCAGCTTTACGCCCACAAGATCATG
Stop **Start** *crtF*
GACATCCCGGCCAGCGCCGAGAGGGGC**TGA**TC**GTG**CCGAAGGACGACCACACG
GGCGCGACGGCCGACCGGACCGCGCAGCCGACAGGAACGGGAAAGCAGCCGCT
GGTTCCGGGCCAGCCCGGGGCGGCGCCGGTGCAGCCGGGGCGGGTGAATTTCTTC
ACCCGGATCGCGCTGTCGCAACGGCTGCATGAAATCTTCGAACGCCTGCCGCTGA
TGAACCGCGTCACCCGGCGCGAGGGCGAGGCGCTCTTCGACATCGTTTCGGGCTT
CGTGCAAAGCCAGGTTCTCTTGGCGATCGTCGAATTCCGGGTGCTGCATATTCTG
GCCGGGGCCTCTTGGCCCTTGCCGCAACTGGCCGAACGCACCGGCCTGGCCGAGG
ACCGGCTGGCGGTGCTGATGCAGGCCGCCGCCGCCTTGAAGCTGGTGAAATTCCG
CCGCGGTCTGTGGCAGCTTGCCCCGCGTGGCGCCGCCTTCATCACCGTGCCAGGG
CTCGAGGCGATGGTGCGCCATCACCCCGTCCTTTACCGCGATCTGGCCGATCCGG
TGGCTTTTCTGAAAGGCGACATCGAACCCGAGCTGGCGGGCTTCTGGCCCTATGT
CTTCGGGCCGCTGGCGCAGGAAGATGCGGGGCTCGCCGAGCGCTATTCGCAGCTG
ATGGCCGACAGCCAGCGCGTCGTGGCCGATGACACCTTGCGGCTTGTCGATCTGC
GCGATGCCAAGCGGGTGATGGATGTGGGCGGCGGCACCGGGGCCTTCCTGCGCG
TCGTGGCCAAGCTTTACCCCGAGCTGCCCTTGACGCTGTTCGACCTGCCGCATGTG
CTGTCGGTGGCGGACCGCTTCAGCCCGAAGCTCGATTTCGCGCCGGGCAGCTTCC
GCGACGATCCGATCCCGCAGGGCGCCGATGTCATCACTTTGGTGCGCGTGCTGTA
TGACCATCCTGACAGCGTCGTCGAACCGCTTCTGGCCAAGGTGCATGCCGCCTTG
CCGCCGGGCGGGCGTCTGATCATCTCGGAGGCGATGGCGGGGGGCGCAAAACCC
GACCGTGCCTGCGATGTCTATTTCGCCTTCTACACGATGGCGATGAGTTCGGGGC
GCACGCGTTCCCCCGAAGAGATCAAGCAAATGCTTGAAAAAGCTGGGTTCACCA
AGGTGTCGAAACCGCGGACCCTGCGCCCCTTCATCACCTCGGTGATCGAGGCCGA
ACGCGGC**TGA**
Stop

Determination of photocaged compound purity by qNMR

Table S2: Compound purities determined by qNMR.

Compound	Purity [%]
NP-cIPTG	88.9 ± 3.4
BEC-cIPTG	90.7 ± 1.3
BC-cIPTG	79.6 ± 1.8

The spectral and (photo-)chemical properties (solubility, absorption maximum, molar extinction coefficient, uncaging quantum yield, uncaging half-life time) of the photocaged compounds NP-cIPTG, BEC-cIPTG and BC-cIPTG have been reported previously [10].

References

1. Hanahan, D. Studies on transformation of *Escherichia coli* with plasmids. *J. Mol. Biol.* **1983**, *166*, 557–580, doi:10.1016/S0022-2836(83)80284-8.

2. Simon, R.; Priefer, U.; Pühler, A. A Broad host range mobilization system for *in vivo* genetic engineering: transposon mutagenesis in gram-negative bacteria. *Bio/Technology* **1983**, *1*, 784–791, doi:10.1038/nbt1183-784.

3. Strnad, H.; Lapidus, A.; Paces, J.; Ulbrich, P.; Vlcek, C.; Paces, V.; Haselkorn, R. Complete genome sequence of the photosynthetic purple nonsulfur bacterium *Rhodobacter capsulatus* SB1003. *J. Bacteriol.* **2010**, *192*, 3545–6, doi:10.1128/JB.00366-10.

4. Hage-Hülsmann, J.; Metzger, S.; Wewer, V.; Buechel, F.; Troost, K.; Thies, S.; Loeschcke, A.; Jaeger, K.-E.; Drepper, T. Biosynthesis of cycloartenol by expression of plant and bacterial oxidosqualene cyclases in engineered *Rhodobacter capsulatus*. *J. Biotechnol.* **2019**, *4*, 100014, doi:10.1016/j.btecx.2020.100014.

5. Binder, D.; Grünberger, A.; Loeschcke, A.; Probst, C.; Bier, C.; Pietruszka, J.; Wiechert, W.; Kohlheyer, D.; Jaeger, K.-E.; Drepper, T. Light-responsive control of bacterial gene expression: precise triggering of the *lac* promoter activity using photocaged IPTG. *Integr. Biol.* **2014**, *6*, 755–765, doi:10.1039/C4IB00027G.

6. Sambrook, J.; Fritsch, E.F.; T. Maniatis *Molecular cloning : a laboratory manual.*; Cold Spring Habor Laboratory Press, New York., **1989**.

7. Salis, H. M.; Mirsky, E. A.; and Voigt, C. A. *Automated design of synthetic ribosome binding sites to control protein expression*. Nat. Biotechnol. **2009**, 27, 946–950. doi:10.1038/nbt.1568.

8. Zhang, J.-Y., Esrom, H., and Boyd, I. W. UV intensity measurement of 308 nm excimer lamp using chemical actinometer. *Appl. Surf. Sci.* **1999**;138–139, 315–319. doi:10.1016/S0169-4332(98)00412-7.

9. Hilgers, F.; Habash, S.S.; Loeschcke, A.; Ackermann, Y.S.; Neumann, S.; Heck, A.; Klaus, O.; Hage-Hülsmann, J.; Grundler, F.M.W.; Jaeger, K.-E.; Schleker, A.S.S.; Drepper, T. Heterologous production of β-caryophyllene and evaluation of its activity against plant pathogenic fungi. *Microorganisms*. **2021**;9. doi:10.3390/microorganisms9010168

10. Hogenkamp, F.; Hilgers, F.; Knapp, A.; Klaus, O.; Bier, C.; Binder, D.; Jaeger, K. E.; Drepper, T. & Pietruszka, J. Effect of photocaged isopropyl β-D-1-thiogalactopyranoside solubility on the light responsiveness of LacI-controlled expression systems in different bacteria. *ChemBioChem*. **2021**;22: 539–547. doi:10.1002/cbic.202000377
